# Sequential action of JNK genes establishes the embryonic left-right axis

**DOI:** 10.1242/dev.200136

**Published:** 2022-05-03

**Authors:** Christopher J. Derrick, Adrian Santos-Ledo, Lorraine Eley, Isabela Andhika Paramita, Deborah J. Henderson, Bill Chaudhry

**Affiliations:** Biosciences Institute, Faculty of Medical Sciences, Newcastle University, International Centre for Life, Central Parkway, Newcastle upon Tyne NE1 3BZ, UK

**Keywords:** JNK, Laterality, PCP, Zebrafish, Kupffer's vesicle, Cilia

## Abstract

The establishment of the left-right axis is crucial for the placement, morphogenesis and function of internal organs. Left-right specification is proposed to be dependent on cilia-driven fluid flow in the embryonic node. Planar cell polarity (PCP) signalling is crucial for patterning of nodal cilia, yet downstream effectors driving this process remain elusive. We have examined the role of the JNK gene family, a proposed downstream component of PCP signalling, in the development and function of the zebrafish node. We show *jnk1* and *jnk2* specify length of nodal cilia, generate flow in the node and restrict *southpaw* to the left lateral plate mesoderm. Moreover, loss of asymmetric *southpaw* expression does not result in disturbances to asymmetric organ placement, supporting a model in which nodal flow may be dispensable for organ laterality. Later, *jnk3* is required to restrict *pitx2c* expression to the left side and permit correct endodermal organ placement. This work uncovers multiple roles for the JNK gene family acting at different points during left-right axis establishment. It highlights extensive redundancy and indicates JNK activity is distinct from the PCP signalling pathway.

## Abstract (precis)

**Summary:** Different members of the JNK gene family play sequential roles in establishing the left-right axis through early regulation of motile cilia and later restriction of *pitx2c* expression.

## INTRODUCTION

Vertebrates exhibit external symmetry; however, many internal organs have asymmetric positioning that requires the establishment of a midline left-right axis in early development (Blum and Ott, 2018; Grimes and Burdine, 2017). Disruption to this axis can result in abnormal organ positioning and, when associated with congenital heart malformations, is known as heterotaxy (Kennedy et al., 2007; Shiraishi and Ichikawa, 2012). The embryonic node, a transient ciliated cavity or pit is believed to be essential for the establishment of left-right identity. Within the node, rotation of nodal cilia generates right-to-left fluid flow (nodal flow) (Amack, 2014; Nonaka et al., 1998), leading to asymmetric expression of the TGFβ family member *Nodal* in the left lateral plate mesoderm (LPM) (Tabin, 2005). *Nodal* expression is self-promoting (Yamamoto et al., 2003) and concomitantly activates its own inhibitors, in particular *Lefty1* in the midline, which prevents Nodal signalling propagating into the right LPM (Meno et al., 1998; Nakamura et al., 2006). Downstream of *Nodal*, the highly conserved transcription factor *Pitx2* (Shiratori et al., 2001) is expressed in the left LPM overlapping multiple organ analgen that undergo asymmetric morphogenesis (Campione et al., 1999). The *Pitx2* gene locus generates two distinct mRNAs, *Pitx2a* and *Pitx2c* that perform subtly different roles in development (Essner et al., 2000). Left-right axis establishment is well conserved in zebrafish, where the function of the node, or Kupffer's vesicle (KV) (Essner et al., 2005; Kupffer, 1868), is proposed to promote left-sided expression of the *Nodal* homolog *southpaw* (*spaw*) (Long et al., 2003). Zebrafish abdominal organs are asymmetrically positioned, with left-sided positioning of the liver and right-sided positioning of the stomach, pancreas and spleen (Horne-Badovinac et al., 2003). In the heart, the first evidence of morphological left-right identity is manifested as jogging: the extension of the linear heart tube under the left eye (Chen et al., 1997; Smith and Uribe, 2021). Dextral heart looping establishes the asymmetric placement of the single atrium and ventricle.

Within the node, motile cilia are positioned distally on each cell (Nonaka et al., 2005), a patterning governed by the highly conserved Wnt planar cell polarity (PCP) pathway (Hashimoto et al., 2010; Minegishi et al., 2017). This pathway also plays crucial roles in regulating polarised cellular behaviours that drive convergent-extension movements during gastrulation (Roszko et al., 2009). Thus, mutations in classical PCP components result in a striking convergent-extension phenotype, seen in zebrafish as shortening of the antero-posterior axis (Jessen et al., 2002; Park and Moon, 2002). Loss of key components of the PCP pathway also impact node development. Vangl2 plays a role in defining the size of the node and positioning nodal cilia, whereas *rock2b* establishes their asymmetric antero-posterior arrangement (Borovina et al., 2010; Wang et al., 2011). Proposed downstream effectors of PCP signalling are the highly conserved Jun N-terminal kinases (JNKs) (Boutros et al., 1998; Moriguchi et al., 1999; Riesgo-Escovar et al., 1996): Ser/Thr kinases that are members of the mitogen activated protein kinase (MAPK) superfamily activated through a MAPKKK, MAPKK and MAPK phosphorylation cascade (Davis, 2000). Vertebrates have three JNK genes: *Jnk1*, *Jnk2* and *Jnk3* (Gupta et al., 1996), *Jnk1* and *Jnk2* are ubiquitously expressed, whilst *Jnk3* expression is restricted to specific structures (Davis, 2000; Santos-Ledo et al., 2020). Double *Jnk1/Jnk2* knockout mouse mutants are embryonic lethal between embryonic day (E) 11 and E12, whereas *Jnk1/Jnk3* or *Jnk2/Jnk3* double mutants are reported to be healthy, demonstrating redundancy and suggesting that genetic compensation could be active in the JNK gene family, but also questioning a direct role for JNK in the PCP pathway (Kuan et al., 1999). Similarly, our recent studies using zebrafish mutants do not implicate the duplicated *jnk1a* and *jnk1b* genes (*mapk8a* and *mapk8b* – ZFIN) in either the PCP pathway or left-right axis specification (Santos-Ledo et al., 2020). In contrast, other studies using antisense morpholino oligonucleotides have proposed that *jnk1* is required for specifying left-right axis through correct nodal cilia length and suggested *jnk1* morphants display defects in heart jogging (Gao et al., 2017). More generally, shorter nodal cilia are widely reported to impact on left-right asymmetry (Gao et al., 2017; Heigwer et al., 2020; Jacinto et al., 2021; Lopes et al., 2010; Neugebauer et al., 2009; Schottenfeld et al., 2007; Yamauchi et al., 2009). Separately, experiments in *Xenopus* have suggested a cooperative role for JNK in the PCP pathway (Kim and Han, 2005; Yamanaka et al., 2002).

Using zebrafish to investigate the link between node function and organ asymmetry (Smith and Uribe, 2021), we set out to address whether a role exists for members of the JNK family in regulating left-right axis development and whether genetic compensation between JNK family members may obscure PCP functions. Generating stable mutants, we characterised the impact of loss of the four zebrafish JNK genes on nodal cilia development and the subsequent result on organ asymmetry. We identify that *jnk1a*, *jnk1b* and *jnk2* (*mapk9* – ZFIN) function non-redundantly in the embryonic node to specify nodal cilia length and are required for directional nodal flow. We show that compromised KV function following loss of *jnk1a*, *jnk1b* and *jnk2*, although disrupting lateralised expression of *spaw,* does not result in abnormal organ asymmetry, defining an early, yet dispensable, role for *jnk1* and *jnk2* in left-right axis establishment. We also identify a novel, later requirement for *jnk3* (*mapk10* – ZFIN) in restricting *pitx2c* expression and promoting lateralised endodermal organ placement.

## RESULTS

We have previously reported that maternal zygotic (MZ) *jnk1a* (*MZjnk1a*), *MZjnk1b* and *MZjnk1a;MZjnk1b* zebrafish mutants display no evidence of left-right disturbance with regard to heart development (Santos-Ledo et al., 2020). However, a morpholino-based study suggested a role for *jnk1* in regulating nodal cilia length (Gao et al., 2017). Therefore, we set out to characterise the impact of loss of JNK genes on left-right axis development in zebrafish.

### *jnk1a* and *jnk1b* specify length of nodal cilia and are required for nodal flow in KV

**Fig. 1.**
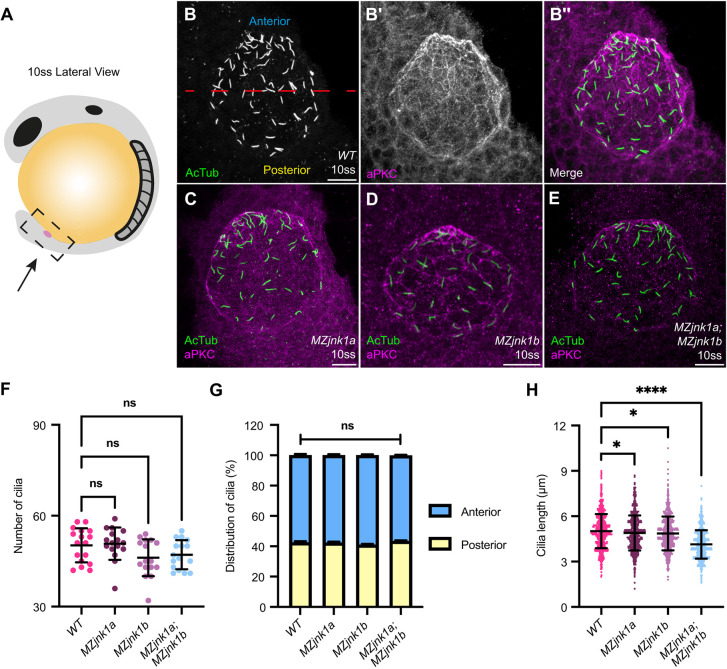
***jnk1a* and *jnk1b* function redundantly to regulate cilia length in the zebrafish left-right organiser.** (A) Schematic of zebrafish embryo at the 10-somite stage (ss), lateral view. Kupffer's vesicle (KV, magenta) is located at the caudal tip of the notochord. The caudal region of the embryo imaged in B-E is outlined. (B,B′) Representative image of wild-type KV at 10 ss used for characterisation of cilial parameters by immunohistochemistry for acetylated tubulin with depiction of anterior (blue) and posterior (yellow) in KV (B) and aPKC (B′). (B″) Merged images. (C-E) Representative *MZjnk1a* (C), *MZjnk1b* (D) and *MZjnk1a;MZjnk1b* (E) KVs at 10 ss labelling acetylated tubulin (green) and aPKC (magenta). (F) Quantification of the number of cilia in KV in wild-type, *MZjnk1a, MZjnk1b* and *MZjnk1a;MZjnk1b* embryos at 10 ss. *jnk1* mutants display no differences in the number of nodal cilia in KV. (G) Quantification of nodal cilia distribution in wild-type, *MZjnk1a, MZjnk1b* and *MZjnk1a;MZjnk1b* embryos at 10 ss. Antero-posterior distribution nodal cilia is unaffected in *jnk1* mutants. (H) Quantification of length of nodal cilial in wild-type, *MZjnk1a, MZjnk1b* and *MZjnk1a;MZjnk1b* embryos at 10 ss. *MZjnk1a* and *MZjnk1b* mutant embryos display a similar significant reduction in cilia length. Loss of both *jnk1a* and *jnk1b* results in a more dramatic reduction in cilia length. (F) Data are mean±s.d., one-way ANOVA, multiple comparisons. Wild type, *n*=17; *MZjnk1a*, *MZjnk1b* and *MZjnk1a;MZjnk1b*, *n*=16. (G) Data are mean±s.e.m., two-way ANOVA, multiple comparisons, *n*=16. (H) Data are mean±s.d., one-way ANOVA, multiple comparisons. Wild type, *n*=809; *MZjnk1a*, *n*=812; *MZjnk1b*, *n*=743; *MZjnk1a;MZjnk1b*, *n*=754. ns, not significant; *P<0.05, ****P<0.0001. (B-E) Anterior is upwards, left is leftwards. Scale bars: 20 µm.

**Fig. 2.**
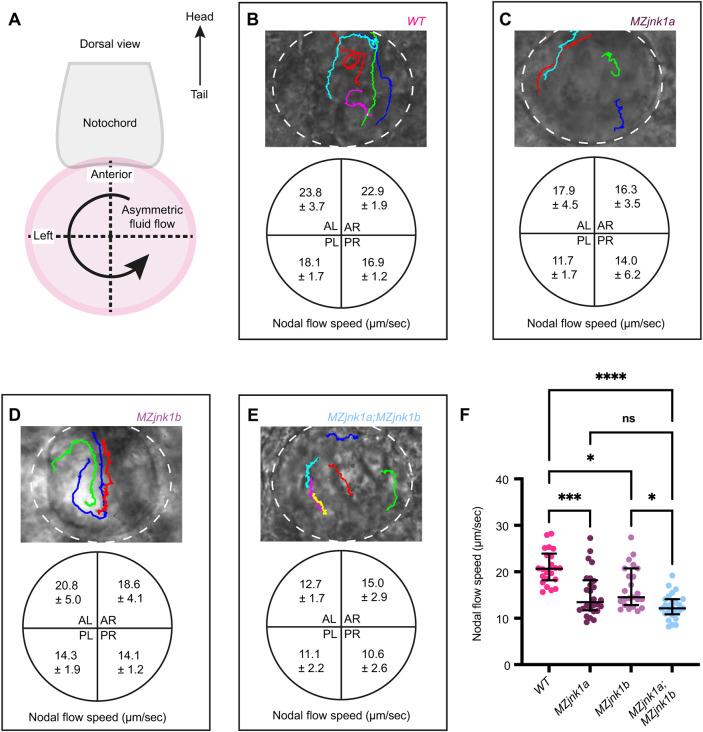
***jnk1a* and *jnk1b* are required for fluid flow in KV.** (A) Schematic showing KV (magenta) at the base of the notochord (grey) and direction of nodal flow (arrow) at 10-14 ss. The notochord was used to define the antero-posterior and left-right axes for subdivision of KV into quadrants. (B-E) Representative bright-field images of wild type (B), *MZjnk1a* (C), *MZjnk1b* (D) and *MZjnk1a;MZjnk1b* KVs (white dotted line), and traces of bead trajectories over time (coloured lines), together with mean bead speed (µm/s) in each quadrant±s.d. (F) Quantification of average bead speed in KV between 10 and 14 ss. *MZjnk1a* and *MZjnk1b* mutant embryos have significantly slower average nodal flow compared with wild-type controls. Loss of both *jnk1a* and *jnk1b* results in a greater reduction in average speed. (F) Data are median±interquartile range, Kruskal-Wallis test, multiple comparisons. Wild type, *n*=22 beads across six embryos; *MZjnk1a*, *n*=26 beads across four embryos; *MZjnk1b*, *n*=23 beads across three embryos; *MZjnk1a;MZjnk1b*, *n*=25 beads across four embryos. (B-E) Anterior is upwards; left is leftwards. ns, not significant. *P<0.05, ***P<0.001, ****P<0.0001. AL, anterior left; AR, anterior right; PL, posterior left; PR, posterior right.

We first examined the impact of loss of *jnk1* activity on KV size using whole-mount mRNA *in situ* hybridisation for *dand5* (*DAN domain family member 5*, formerly *charon*) at the 8-somite stage (ss) (Fig. S1A), showing comparable KV size between wild type, *MZjnk1a*, *MZjnk1b* and *MZjnk1a;MZjnk1b* mutants (Fig. S1B). We next characterised number, distribution and length of cilia within KV at 10 ss using immunohistochemistry (Fig. 1A-E). Although total cilia number and antero-posterior distribution were unaffected in any *MZjnk1*-null mutants (Fig. 1F,G), both *MZjnk1a* and *MZjnk1b* mutants displayed a subtle decrease in the length of nodal cilia (2.6% and 3.1%, respectively) (Fig. 1H). However, in *MZjnk1a;MZjnk1b* double mutants, there was a 17.6% reduction in cilia length, demonstrating redundancy of these *jnk1* paralogues in regulating KV cilial length (Fig. 1H). Surprisingly, despite a relatively minor reduction in cilial length, there were significant reductions in the counter-clockwise nodal flow in both *MZjnk1a* and *MZjnk1b* mutant embryos (Fig. 2C,D,F, Movies 1-3), and a greater reduction in KV flow in *MZjnk1a;MZjnk1b* mutants (Fig. 2E,F, Movie 4), potentially indicating reduction in cilia motility as well as length.

### Normal organ positioning despite disturbed *southpaw* expression in *jnk1* mutants

**Fig. 3.**
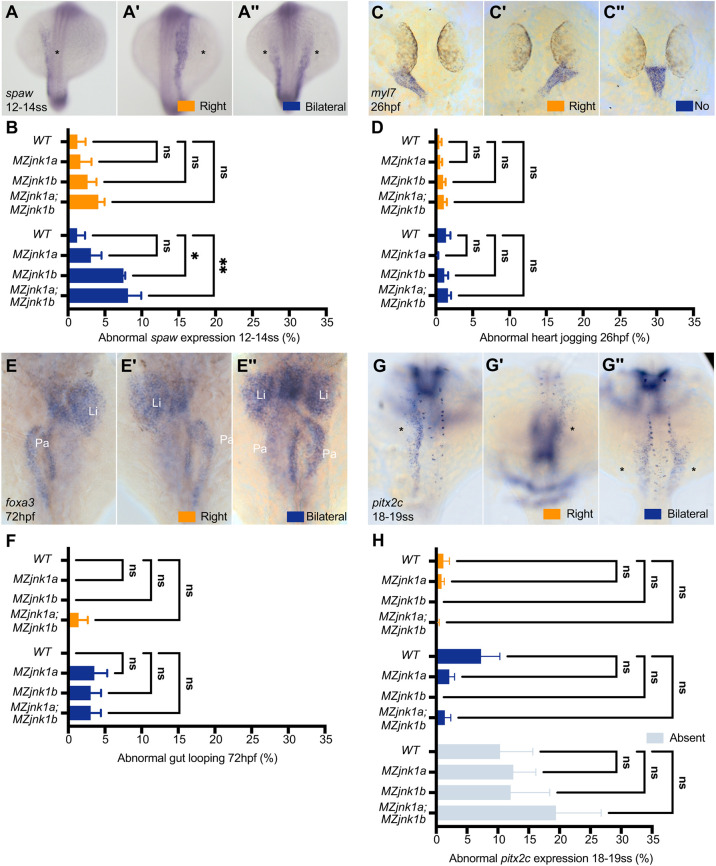
**Loss of *jnk1* disrupts Nodal signalling, but not organ asymmetry.** (A-A″) Representative images of mRNA *in situ* hybridisation for the zebrafish *Nodal* homolog *southpaw* (*spaw*) at 12-14 ss, showing normal expression in the left lateral plate mesoderm (LPM) (A, asterisk), and abnormal right-sided (A′, asterisk) or bilateral (A″, asterisks) expression. (B) Characterisation of abnormal *spaw* expression in wild type, and in *MZjnk1a*, *MZjnk1b* and *MZjnk1a;MZjnk1b* mutants at 12-14 ss. *MZjnk1b* and *MZjnk1a;MZjnk1b* mutants display a significant increase in the percentage of embryos with bilateral *spaw* expression. Loss of *jnk1a* alone does not have a significant impact on *spaw* expression. (C-C″) Representative images of mRNA *in situ* hybridisation of the pan-cardiac marker *myosin, light chain 7, regulatory* (*myl7*) at 26 hpf showing normal left jogging of the heart (C), abnormal right jogging (C′) or no jogging (C″). (D) Quantification of jogging in wild type, and *MZjnk1a*, *MZjnk1b* and *MZjnk1a;MZjnk1b* mutants at 26 hpf. Loss of *jnk1* does not affect heart jogging. (E-E″) Representative images of mRNA *in situ* hybridisation of the endodermal marker *forkhead box A3* (*foxa3*) at 72 hpf showing organ placement of the liver (Li) and pancreas (Pa) following gut looping (E), reversed gut looping (E′) or a failure of LPM migration, resulting in a bilateral gut, most obviously observed by the presence of bilaterally positioned livers (E″). (F) Quantification of gut looping in wild type, and *MZjnk1a*, *MZjnk1b* and *MZjnk1a;MZjnk1b* mutants at 72 hpf. Loss of *jnk1* does not affect endoderm morphogenesis. (G-G″) Representative images of mRNA *in situ* hybridisation of *paired-liked homeodomain 2, isoform c* (*pitx2c*) at 18-19 ss, showing normal expression in the left lateral plate mesoderm (F, asterisk), and abnormal right-sided (F′, asterisk) or bilateral (F″, asterisks) expression. (H) Characterisation of abnormal *pitx2c* expression in wild type, and *MZjnk1a*, *MZjnk1b* and *MZjnk1a;MZjnk1b* mutants at 18-19 ss. Loss of *jnk1* does not result in abnormal expression of *pitx2c*. (B) Data are mean±s.e.m., two-way ANOVA comparison of right and bilateral. *n*=3 clutches. Minimum clutch sizes: wild type, *n*=27; *MZjnk1a*, *n*=21; *MZjnk1b*, *n*=27; *MZjnk1a;MZjnk1b*, *n*=17. (D) Data are mean±s.e.m., two-way ANOVA comparison of right jogging and no jogging. *n*=7 clutches for wild type, *n*=6 clutches for *MZjnk1a*, and *n*=8 clutches for *MZjnk1b* and *MZjnk1a;MZjnk1b*. Minimum clutch sizes: wild type, *n*=32; *MZjnk1a*, *n*=46; *MZjnk1b*, *n*=46; *MZjnk1a;MZjnk1b*, *n*=43. (F) Data are mean±s.e.m., two-way ANOVA comparison of right and bilateral. *n*=3 clutches. Minimum clutch sizes: wild type, *n*=25, *MZjnk1a*, *n*=16; *MZjnk1b*, *n*=21; *MZjnk1a;MZjnk1b*, *n*=22. (H) Data are mean±s.e.m., two-way ANOVA, multiple comparisons analysing right, bilateral and absent *pitx2c* expression. *n*=5 clutches. Minimum clutch sizes: wild type, *n*=75; *MZjnk1a*, *n*=73; *MZjnk1b*, *n*=64; *MZjnk1a:MZjnk1b*, *n*=37. (A-A″,C-C″,G-G″) Dorsal views. (E-E″) Ventral views. ns, not significant. *P<0.05, **P<0.01.

Leftward flow within KV is proposed to drive left-sided expression of *spaw* (Long et al., 2003; Yamamoto et al., 2003). We therefore examined *spaw* expression at 12-14 ss in our *MZjnk1* mutants (Fig. 3A,B) to determine the effect of altered nodal flow. Despite a marked reduction in KV flow (Fig. 2F), there was no significant increase in isolated right-sided *spaw* expression (Fig. 3B) but there were increases in the penetrance of bilateral *spaw* expression in *MZjnk1b* and *MZjnk1a;MZjnk1b* mutants, suggesting that loss of *jnk1b* disrupts KV function (Fig. 3B). We then investigated whether abnormal *spaw* expression would translate into abnormal organ positioning. Left-sided expression of *spaw* is required for leftward heart jogging at 1 dpf (day post-fertilisation) (Lenhart et al., 2013; Noël et al., 2013; Smith et al., 2008; Veerkamp et al., 2013) (Fig. 3C) and for the asymmetric movements of the LPM, positioning the liver on the left and the pancreas on the right of the midline (Horne-Badovinac et al., 2003; Yin et al., 2010) (Fig. 3E). In keeping with correct lateralised *spaw* expression, heart jogging and liver/pancreas positioning was normal in *MZjnk1a* mutants (Fig. 3D,F). However, despite the increase in bilateral *spaw* expression in *MZjnk1b* and *MZjnk1a;MZjnk1b* mutants (Fig. 3B), there was no disturbance of heart jogging or abdominal organ placement (Fig. 3D,F). To investigate the uncoupling between *spaw* laterality and organ asymmetry in *jnk1* mutants, we examined the expression of the highly conserved Nodal-target gene *paired-liked homeodomain 2* (*pitx2*) in the LPM at 18-19 ss (Fig. 3G). Although we observed predominantly left-sided expression of the *pitx2c* splice-form in wild type, we were surprised to see high frequencies of absent and bilateral expression (Fig. 3H). Expression of *pitx2c* in *MZjnk1a*, *MZjnk1b* and *MZjnk1a;MZjnk1b* was comparable with wild-type controls and no increase in bilateral *pitx2c* expression was observed (Fig. 3H).

Taken together, these data show that *jnk1* is required for the specification of cilial length, for nodal flow in KV and for lateralised *spaw* expression. However, bilateral *spaw* expression in *jnk1* mutants does not translate into bilateral *pitx2c* expression or into abnormal organ placement, suggesting other factors acting parallel to the KV axis could be functioning to drive asymmetric organ positioning or that other genes function redundantly with *jnk1* to establish laterality.

### *jnk2* acts with *jnk1a* and *jnk1b* to regulate early left-right axis development

**Fig. 4.**
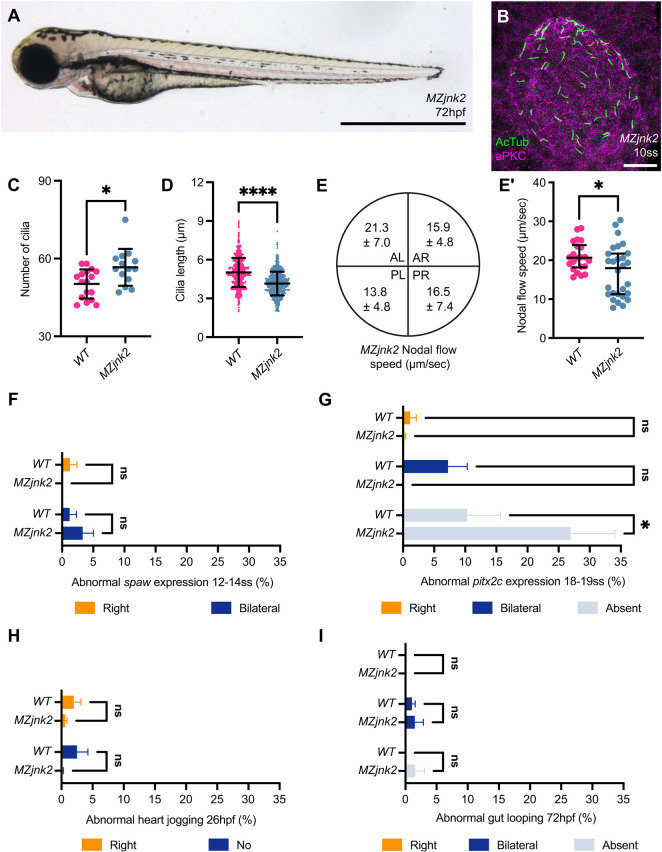
***jnk2* regulates nodal cilia development and KV flow.** (A) Representative bright-field image of *MZjnk2* mutant at 3 dpf. (B) Representative *MZjnk2* KV at 10 ss labelling acetylated tubulin (green) and aPKC (magenta). (C) Quantification of number of cilia in wild-type and *MZjnk2* embryos at 10 ss. Loss of *jnk2* results in a significant increase in the number of nodal cilia. (D) Quantification of length of nodal cilial in wild-type and *MZjnk2* embryos at 10 ss. *MZjnk2* mutant embryos have a significant reduction in the length of nodal cilia. (E,E′) Quantification of nodal flow speed in *MZjnk2* by quadrant (μm/s; data are mean±s.d.) (E) and average speed (E′) between 10 and 14 ss. Loss of *jnk2* results in a significant reduction in average speed (E′). (F) Loss of *jnk2* does not impact normal *spaw* expression at 12-14 ss. (G) Characterisation of abnormal *pitx2c* expression in wild type and *MZjnk2* mutants at 18-19 ss. Loss of *jnk2* results in a significant proportion of embryos that do not have *pitx2c* expression in the lateral plate mesoderm. (H) Heart jogging is unaffected in *MZjnk2* mutants. (I) Gut looping occurs normally in *MZjnk2* mutants. (C) Data are mean±s.d., Welch's *t*-test. *MZjnk2*, *n*=14. Wild-type data are from Fig. 1F. (D) Data are mean±s.d., Welch's *t*-test. *MZjnk2*, *n*=737. Wild-type data are from Fig. 1H. (E′) Data are median±interquartile range, Mann–Whitney test. *MZjnk2*, *n*=28 beads from six embryos. Wild-type data are from Fig. 2F. (F) Data are mean±s.e.m., two-way ANOVA comparison of right and bilateral. *n*=3 clutches. Minimum clutch size: *MZjnk2*, *n*=24. Wild-type data are from Fig. 3B. (G) Data are mean±s.e.m., two-way ANOVA, multiple comparisons analysing right, bilateral and absent. *n*=5 clutches. Minimum clutch size: *MZjnk2*, *n*=81. Wild-type data are from Fig. 3H. (H) Data are mean±s.e.m., two-way ANOVA comparison of right and no jog. *n*=6 clutches for wild type and *MZjnk2*. Minimum clutch sizes: wild type, *n*=101; *MZjnk2*, *n*=46. (I) Data are mean±s.e.m., two-way ANOVA comparison of right and bilateral. *n*=3 clutches. Minimum clutch sizes: wild type, *n*=98; *MZjnk2*, *n*=89. (A) Lateral view, anterior leftwards. Scale bar: 1 mm. (B) Anterior upwards. Scale bar: 20 µm. ns, not significant. *P<0.05, ****P<0.0001. AL, anterior left, AR, anterior right; PL, posterior left; PR, posterior right.

Members of the JNK gene family, particularly *Jnk1* and *Jnk2* have been suggested to compensate for one another (Kuan et al., 1999). We therefore generated *jnk2* mutants using CRISPR-Cas9 genome editing to investigate whether compensation was occurring within KV (Fig. S2A,B). As with *MZjnk1* mutants, *MZjnk2* mutants were fertile and appear morphologically normal (Fig. 4A). We first characterised the impact of loss of *jnk2* on KV structure and function, noting a small increase in KV size associated with increased number of nodal cilia (Fig. S2C, Fig. 4C). Although normally distributed across the KV (Fig. S2D), *MZjnk2* mutants had significantly shorter nodal cilia (17.3%) (Fig. 4D), similar to *MZjnk1a;MZjnk1b* mutants (Fig. 1H). Although *MZjnk2* mutants maintain a counter-clockwise nodal flow that is reduced (Fig. 4E,E′, Movie 5), this was small and not in keeping with dramatic changes seen in the *MZjnk1* mutants, suggesting cilial motility might be additionally impaired in the *MZjnk1* mutants. Confirming a minimal impact on KV function, *spaw* expression was normal (Fig. 4F), but, surprisingly, there was a significant increase in the proportion of *MZjnk2* embryos without *pitx2c* expression in the LPM (Fig. 4G). However, despite this, heart and abdominal organ placement was normal (Fig. 4H,I).

**Fig. 5.**
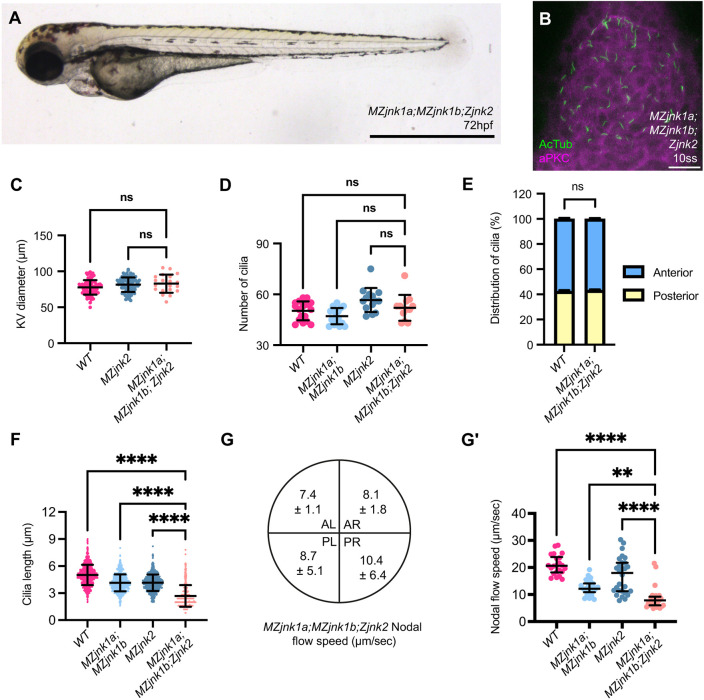
***jnk1a, jnk1b* and *jnk2* function together in nodal cilia development and KV function.** (A) Representative bright-field image of *MZjnk1a;MZjnk1b;Zjnk2* embryo at 3 dpf. (B) Representative *MZjnk1a;MZjnk1b;Zjnk2* KVs at 10 ss labelling acetylated tubulin (green) and aPKC (magenta). (C) KV diameter quantification in wild type, and in *MZjnk2* and *MZjnk1a;MZjnk1b;Zjnk2* mutant embryos at 8 ss. KV size is not impacted by loss of *jnk1* and *jnk2* function. (D) Quantification of number of cilia in wild type, and in *MZjnk1a;MZjnk1b*, *MZjnk2* and *MZjnk1a;MZjnk1b;Zjnk2* mutant embryos at 10 ss. Loss of *jnk1a*, *jnk1b* and *jnk2* does not impact cilia number in KV. (E) Quantification of nodal cilia distribution in wild-type and *MZjnk1a;MZjnk1b;Zjnk2* embryos at 10 ss. Antero-posterior distribution of nodal cilia is unaffected by loss of *jnk2* in a *jnk1*-null background. (F) Quantification of length of nodal cilial in wild type, and in *MZjnk1a;MZjnk1b*, *MZjnk2* and *MZjnk1a;MZjnk1b;Zjnk2* mutant embryos at 10 ss. *MZjnk1a;MZjnk1b;Zjnk2* mutant embryos have a greater reduction in the length of nodal cilia compared with *MZjnk1a;MZjnk1b* or *MZjnk2* mutants. (G,G′) Quantification of nodal flow speed in *MZjnk1a;MZjnk1b;Zjnk2* by quadrant (µm/s; data are mean±s.d.) (G) and average speed (G′) between 10 and 14 ss. *MZjnk1a;MZjnk1b;Zjnk2* mutant embryos have a greater reduction in the average speed of nodal flow compared with *MZjnk1a;MZjnk1b* or *MZjnk2* mutants. (C) Data are mean±s.d., one-way ANOVA, multiple comparisons. *MZjnk1a;MZjnk1b;Zjnk2*, *n*=20. Wild-type data are from Fig. S1B. *MZjnk2* data are from Fig. S2C. (D) Data are mean±s.d., one-way ANOVA, multiple comparisons. *MZjnk1a;MZjnk1b;Zjnk2*, *n*=12. Wild-type and *MZjnk1a;MZjnk1b* data are from Fig. 1F. *MZjnk2* data are from Fig. 4C. (E) Data are mean±s.e.m., two-way ANOVA, multiple comparisons. *MZjnk1a;MZjnk1b;Zjnk2*, *n*=14. Wild-type data are from Fig. 1G. (F) Data are mean±s.d., one-way ANOVA, multiple comparisons. *MZjnk1a;MZjnk1b;Zjnk2*, *n*=466. Wild-type and *MZjnk1a;MZjnk1b* data are from Fig. 1H. *MZjnk2* data are from Fig. 4D. (G′) Data are median±interquartile range, Kruskal–Wallis test, multiple comparisons, *MZjnk1a;MZjnk1b;Zjnk2*, *n*=28 beads across three embryos. Wild-type and *MZjnk1a;MZjnk1b* data are from Fig. 2F. *MZjnk2* data are from Fig. 4E′. (A) Lateral view, anterior leftwards. Scale bar: 1 mm. (B) Anterior upwards. Scale bar: 20 µm. ns, not significant. **P<0.01, ****P<0.0001. AL, anterior left; AR, anterior right; PL, posterior left; PR, posterior right.

**Fig. 6.**
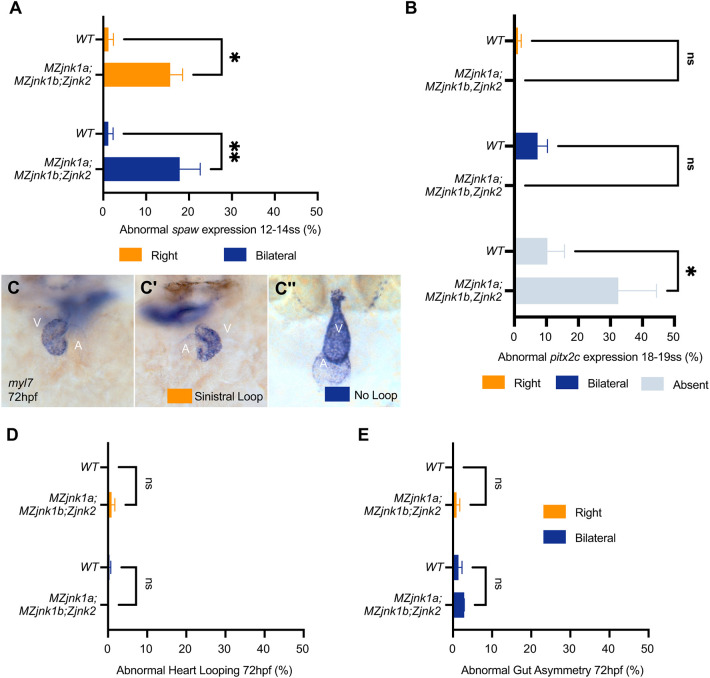
**Asymmetric *spaw* expression is dispensable for organ asymmetry.** (A) Characterisation of abnormal *spaw* expression in wild type and *MZjnk1a;MZjnk1b;Zjnk2* mutants at 12-14 ss. Loss of *jnk1* and *jnk2* results in ∼40% of embryos displaying either right or bilateral *spaw* expression. (B) Characterisation of abnormal *pitx2c* expression in wild type and *MZjnk1a;MZjnk1b;Zjnk2* mutants at 18-19 ss; loss of *jnk1* and *jnk2* results in a significant increase in the proportion of embryos without *pitx2c* expression in the LPM. (C-C″) Representative images of mRNA *in situ* hybridisation for the pan-cardiac marker *myl7* at 72 hpf showing normal dextral looping of the heart (C), abnormal sinistral (reverse) looping (C′) or non-looped hearts (C″). (D,E) Quantification of (D) heart looping and (E) gut looping in wild type and *MZjnk1a;MZjnk1b;Zjnk2* mutants at 72 hpf. Loss of *jnk1* and *jnk2* activity does not impact organ laterality. (A) Data are mean±s.e.m., two-way ANOVA comparison of right and bilateral. *n*=3 clutches. Minimum clutch size: *MZjnk1a;MZjnk1b;Zjnk2*, *n*=10. Wild-type data are from Fig. 3B. (B) Data are mean±s.e.m., two-way ANOVA, multiple comparisons analysing right, bilateral and absent. *n*=5 clutches. Minimum clutch size: *MZjnk1a;MZjnk1b;Zjnk2*, *n*=40. Wild-type data are from Fig. 3H. (D) Data are mean±s.e.m., two-way ANOVA comparison of sinistral and no loop. *n*=3 clutches. Minimum clutch sizes: wild type, *n*=88; *MZjnk1a;MZjnk1b;Zjnk2*, *n*=34. (E) Data are mean±s.e.m., two-way ANOVA comparison of right and bilateral. *n*=3 clutches, same clutches as in D. (C-C″) Ventral view. ns, not significant. *P<0.05, **P<0.01. V, ventricle; A, atrium.

Having identified a partially overlapping role for *jnk2* with *jnk1a* and *jnk1b* in KV, we generated *MZjnk1a;MZjnk1b;Zjnk2* (Z, zygotic) mutants to examine potential redundancy between *jnk1* and *jnk2*. *MZjnk1a;MZjnk1b;Zjnk2* mutants did not display any overt morphological abnormalities, KV size was normal, as was the number and distribution of cilia (Fig. 5A-E). However, there was a 47% reduction in nodal cilial length and a 59% reduction in speed of nodal flow (Fig. 5F-G′) in which injected beads showed no directional movement, demonstrating a loss of KV function (Movie 6). The expression pattern of *spaw* was greatly disturbed in *MZjnk1a;MZjnk1b;Zjnk2* mutants, with 40% exhibiting either right-sided or bilateral expression (Fig. 6A). Despite these severe disturbances, there was no increase in the proportion of embryos that displayed bilateral or right-sided *pitx2c* expression, but instead there was an increase in the proportion of *MZjnk1a;MZjnk1b;Zjnk2* mutants without *pitx2c* expression in the LPM (Fig. 6B).

Having established that loss of *jnk1* and *jnk2* has a dramatic impact on KV function, resulting in disrupted *spaw* expression, we characterised the result of these early defects on organ asymmetry at 72 hpf. Using a combination of *myosin, light chain 7, regulatory* (*myl7*) and *forkhead box A3* (*foxa3*) antisense mRNA probes for whole-mount *in situ* hybridisation, we examined the directionality of heart (Fig. 6C-C″) and gut (Fig. 3E-E″) looping in the same embryo. We did not observe any differences in organ asymmetry in *MZjnk1a;MZjnk1b;Zjnk2* mutants compared with their siblings (heterozygotes or wild type for the *jnk2* mutation) or a wild-type population (Fig. 6D,E). In summary, significant disruption to the lateralised expression of *spaw* and *pitx2c* in *MZjnk1a;MZjnk1b;Zjnk2* mutants does not translate to loss of stereotypical asymmetric organ placement. This suggests that other mechanisms, functioning in parallel to nodal flow, can correctly establish organ asymmetry during early development.

### *jnk3* functions distinctly from other JNK family members in the generation of left-right asymmetry

**Fig. 7.**
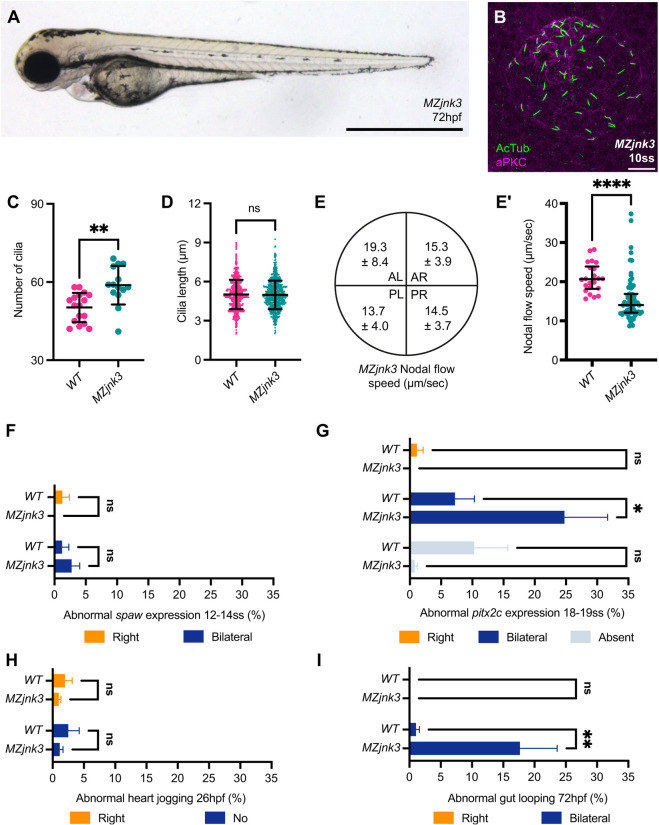
***jnk3* is required to restrict *pitx2c.*** (A) Representative bright-field image of *MZjnk3* mutant at 3 dpf. (B) Representative *MZjnk3* KV at 10 ss labelling acetylated tubulin (green) and aPKC (magenta). (C) Quantification of number of cilia in wild-type and *MZjnk3* embryos at 10 ss. Loss of *jnk3* results in a significant increase in the number of nodal cilia. (D) Quantification of length of nodal cilial in wild-type and *MZjnk3* embryos at 10 ss. Loss of *jnk3* does not affect cilial length. (E,E′) Quantification of nodal flow speed in *MZjnk3* by quadrant (µm/s; data are mean±s.d.) (E) and average speed (E′) between 10 and 14 ss. Loss of *jnk3* results in a significant reduction in average speed (E′). (F) Loss of *jnk3* does not impact normal *spaw* expression at 12-14 ss. (G) Characterisation of abnormal *pitx2c* expression in wild type and *MZjnk3* mutants at 18-19 ss. Loss of *jnk3* leads to a significant increase in the proportion of embryos that display bilateral *pitx2c* expression. (H) Heart jogging is unaffected in *MZjnk3* mutants. (I) *MZjnk3* mutants have a significant proportion of bilaterally positioned abdominal organs. (C) Data are mean±s.d., Welch's *t*-test. *MZjnk3*, *n*=14. Wild-type data are from Fig. 1F. (D) Data are mean±s.d., Welch's *t*-test. *MZjnk3*, *n*=720. Wild-type data are from Fig. 1H. (E′) Data are median±interquartile range, Mann–Whitney test. *MZjnk3*, *n*=61 beads across 11 embryos. Wild-type data are from Fig. 2F. (F) Data are mean±s.e.m., two-way ANOVA comparison of right and bilateral. *n*=3 clutches. Minimum clutch size: *MZjnk3*, *n*=23. Wild-type data are from Fig. 3B. (G) Data are mean±s.e.m., two-way ANOVA, multiple comparisons analysing right, bilateral and absent. *n*=5 clutches. Minimum clutch size: *MZjnk3*, *n*=81. Wild-type data are from Fig. 3H. (H) Data are mean±s.e.m., two-way ANOVA comparison of right and no jog. *n*=8 clutches for *MZjnk3*. Minimum clutch size: *MZjnk3*, *n*=46. Wild-type data are from Fig. 4H. (I) Data are mean±s.e.m., two-way ANOVA comparison of right and bilateral. *n*=3 clutches. Minimum clutch size: *MZjnk3*, *n*=92. Wild-type data are from Fig. 4I. (A) Lateral view, anterior leftwards. Scale bar: 1 mm. (B) Anterior upwards. Scale bar: 20 µm. ns, not significant. *P<0.05, **P<0.01, ****P<0.0001. AL, anterior left; AR, anterior right; PL, posterior left; PR, posterior right.

To complete our analysis, we also generated *jnk3* mutants by CRISPR-Cas9 mutagenesis (Fig. S3A,B). As with all our generated JNK mutants, *MZjnk3* mutants are morphologically normal and fertile (Fig. 7A). Similar to *MZjnk2* mutants, there was an increase in the number of nodal cilia present in KV of *MZjnk3* mutants, but KV size was unaffected (Fig. 7C, Fig. S3C). Cilia length and distribution was normal in *MZjnk3* mutants (Fig. 7D, Fig. S3D). Despite normal cilia length, there was a 25% reduction in speed of nodal flow but, similar to other single JNK mutants, directionality was not affected (Fig. 7E,E′, Movie 7), and the function of KV was sufficient to ensure normal expression of *spaw* in the LPM (Fig. 7F). Strikingly, and in contrast to normal *spaw* expression, 25% of *MZjnk3* mutants showed bilateral *pitx2c* expression (Fig. 7G). This did not impact on heart jogging (Fig. 7H), but did correlate with the abnormal presence of bilateral liver and pancreatic anlagen in 20% of *MZjnk3* embryos (Fig. 7I).

We next generated *MZjnk1a;MZjnk1b;Zjnk3* mutants (Fig. S4A) to further examine potential compensation in the JNK gene family. Loss of zygotic *jnk3* had no impact on the severity of the *MZjnk1a;MZjnk1b* phenotype with respect to cilia length or distribution, and the reduction in KV flow was comparable with *MZjnk1a;MZjnk1b* mutants (Fig. S4B-F′, Movie 8). These findings suggest that *jnk3* may act downstream of *jnk1* in an epistatic mechanism. We also characterised organ looping in *MZjnk1a;MZjnk1b;Zjnk3* mutants and did not observe any significant disturbances to laterality (Fig. S4G,H).

Having established that all JNK genes play a role in generating nodal flow, but to seemingly different extents, we analysed flow patterns within anterior and posterior compartments of KV (Fig. S5). This identified that the main contributing factor to a reduction in flow is disruption to flow in the anterior compartment of KV (Fig. S5A) whereas posterior flow was only significantly affected when both *jnk1a* and *jnk1b* were absent (Fig S5B). This analysis also confirmed that *MZjnk1a;MZjnk1b;Zjnk2* mutants display the most compromised KV function, particularly in the anterior compartment (Fig. S5A).

Although we wished to examine KV structure and function in *MZjnk1a;MZjnk1b;Zjnk2;Zjnk3* mutant embryos, this proved difficult as both *jnk2* and *jnk3* lie on chromosome 21, and breeding between these alleles could only produce heterozygotes in *trans*. Meiotic recombination events that would bring *jnk2* and *jnk3* alleles into *cis* were extremely rare (<1%) and we were not successful in establishing the line.

### *jnk1* and *jnk2* are required to establish the midline barrier

**Fig. 8.**
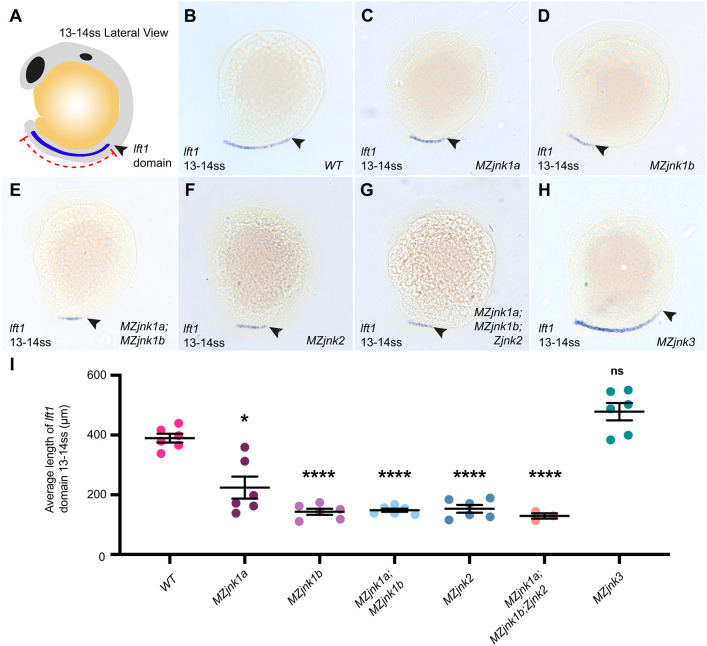
***jnk1* and *jnk2* are required for early establishment of the midline barrier.** (A) Schematic of zebrafish embryo at 13-14 ss. Lateral view. *lefty1* (*lft1*) expression (blue) extends anteriorly from the base of the notochord (red). Arrowhead indicates the anterior limit of expression. Representative images of mRNA *in situ* hybridisation of *lft1* at 13-14 ss in (B) wild type, and (C) *MZjnk1a*, (D) *MZjnk1b*, (E) *MZjnk1a;MZjnk1b*, (F) *MZjnk2*, (G) *MZjnk1a;MZjnk1b;Zjnk2* and (H) *MZjnk3* mutant embryos. Arrowheads indicate the anterior limit of *lft1* expression in the notochord. (I) Quantification of the average length of *lft1* expression at 13-14 ss. Loss of *jnk1a* results in ∼50% reduction in average length of *lft1* domain. *MZjnk1b*, *MZjnk1a;MZjnk1b*, *MZjnk2* and *MZjnk1a;MZjnk1b;Zjnk2* mutants display a more dramatic and similar reduction in the extent of *lft1* propagation in the notochord. *lft1* expression is unaffected in *MZjnk3* mutants. (B-H) Lateral view, anterior is towards the top. (I) Data are mean±s.e.m., Brown-Forsythe and Welch ANOVA, multiple comparisons, with notations denoting result of test between wild type and respective mutant. *n*=6 clutches. Minimum clutch sizes: wild type, *n*=82; *MZjnk1a*, *n*=73; *MZjnk1b*, *n*=61; *MZjnk1a;MZjnk1b*, *n*=78; *MZjnk2*, *n*=59; *MZjnk3*, *n*=69; *MZjnk1a;MZjnk1b;Zjnk2*, *n*=37. (B-H) Lateral views, anterior leftwards. ns, not significant. *P<0.05, ****P<0.0001.

Analysis of left-right specification in JNK mutants identified that bilateral expression of *spaw* is the principal abnormality seen in *MZjnk1a;MZjnk1b;Zjnk2* mutants (Fig. 6A), whereas bilateral *pitx2c* expression is observed in *MZjnk3* mutants (Fig. 7H). This suggested that the midline barrier might be dependent on JNK activity. In wild type at 13-14 ss, *lefty1* (*lft1*) expression extends anteriorly from the caudal region of the embryo, setting up a molecular barrier to maintain left-sided *spaw* expression (Fig. 8A,B). We identified a failure in the propagation of *lft1* in *MZjnk1a*, *MZjnk1b* and *MZjnk1a;MZjnk1b* null embryos (Fig. 8C-E,I), with a less severe reduction in the length of the *lft1* domain in *MZjnk1a* mutants, which correlates with the reduced penetrance of bilateral *spaw* expression (Figs 3B and 8C,I). Surprisingly and despite normal *spaw* expression in the left LPM (Fig. 4G), *MZjnk2* mutants also displayed a failure in anterior propagation of *lft1* along the midline, similar to *MZjnk1b* and *MZjnk1a;MZjnk1b* mutants (Fig. 8F,I). *lft1* propagation is also compromised in *MZjnk1a;MZjnk1b;Zjnk2* mutants, comparably to single *MZjnk1* or *MZjnk2* mutants (Fig. 8G,I). In *MZjnk3* mutants, *lft1* expression is unaffected (Fig. 8H,I), correlating with normal *spaw* expression (Fig. 7G). However, this does not account for the bilateral expression of *pitx2c* in *MZjnk3* (Fig. 7H) and may suggest multiple roles for JNK genes, potentially outside KV in the establishment of laterality.

In conclusion, all JNK family members play overlapping and specific roles in establishing the embryonic left-right axis. *jnk1a*, *jnk1b* and *jnk2* are required for normal nodal cilial length and it appears they may also be important in cilial motility, as *jnk1a*, *jnk1b* and *jnk3* mutants appear to have much greater reductions in KV flow relative to cilial shortening. For *jnk1b*, this translates to abnormal bilateral expression of *spaw*, with an additional effect when *jnk2* is also inactivated. This appears to be related to abnormal midline barrier function, as *lft1* is caudally restricted in these mutants. There is an increase in the proportion of embryos lacking *pitx2c* expression in *MZjnk1a;MZjnk1b;Zjnk2* mutants, but it seems that the mechanisms that maintain left-sided *pitx2c* are intact in these mutants as the frequency of bilateral *pitx2c* expression that might be expected based on *spaw* expression is not seen in these mutants (compare Fig. 6B with Fig. 6A) and asymmetrical organ placement is maintained (Fig. 6C,D). In contrast *jnk3* plays a minor role in KV development and function. Although loss of *jnk3* does not affect cilial length, there is a reduction in KV flow. However, despite this, normal *spaw* expression is established but, surprisingly, there is a marked increase in bilateral *pitx2c* expression (Fig. 7F,G), even though midline barrier function appears normal. Although development of the heart is unaffected, abdominal organ development is strongly affected, with bilateral liver and pancreatic anlagen in *MZjnk3* mutants occurring at a frequency comparable with that of bilateral *pitx2c* expression (Fig. 7H,I).

## DISCUSSION

We have characterised the roles of the JNK genes in establishing left-right asymmetry and show, as previously suggested, that *jnk1* promotes nodal cilial length (Gao et al., 2017), but that this role is shared by *jnk2*; together, both *jnk1* and *jnk2* are required for normal KV function (Fig. 5, Fig. S5). How JNK genes co-ordinate cilia length remains an unresolved issue, as numerous proteins regulate cilia length at the level of post-translational modification. One interesting candidate, the JNK-interacting protein 1 (JIP1), is a regulator of JNK signalling and a cargo protein for the microtubular motor kinesin 1, which is required for axon elongation (Dajas-Bailador et al., 2008). Alternatively, *jnk1* and/or *jnk2* may regulate transcription factors involved in ciliogenesis, such as *rfx3* or *foxj1* (Alten et al., 2012; Bonnafe et al., 2004) in response to developmental signals. In zebrafish, upstream of *foxj1a*, Notch signalling is crucial in regulating nodal cilia length: overactivity increases cilia length, whereas *deltaD* mutants have shorter cilia (40% reduction), a dramatic reduction in nodal flow, and disruption to *spaw* and *pitx2* expression (Lopes et al., 2010). *foxj1* is also regulated by FGF signalling, with *fgfr4* morphants displaying a 26% reduction in cilia length (Gao et al., 2017; Neugebauer et al., 2009). The micro-RNA *miR103/107* appears to regulate ciliogenesis downstream of *foxj1a*, acting on a cohort of genes, including those involved in cilia assembly (Heigwer et al., 2020). *miR103/107* morphants also display shorter cilia (20% reduction) and disrupted asymmetric organ positioning (Heigwer et al., 2020). Importantly, where nodal cilia are either excessively long or significantly shorter, expression of *spaw* and *pitx2* are disrupted, suggesting a window of optimum nodal cilial length (Lopes et al., 2010). Despite a comparably dramatic reduction in nodal cilia length in *MZjnk1a;MZjnk1b;Zjnk2* mutants (47%, Fig. 5) and loss of KV function (Movie 6), organ looping is normal, suggesting that nodal cilia may function redundantly to establish organ laterality (Fig. S6). Of note, whereas *fgfr1* morphants have shortened nodal cilia, they also display a curved body axis, which is a more overt readout of cilial defects, that we did not observe in any JNK mutant (Figs 4, 5 and 7, Fig. S4) (Neugebauer et al., 2009; Santos-Ledo et al., 2020). The absence of such obvious morphological defects in JNK mutants suggests different programmes may generate distinct classes of cilia and that the role of JNK genes may be specific to nodal or, more generally, motile cilia.

Nodal cilia length, orientation and position in the node are crucial for the generation of nodal flow: the proposed symmetry-breaking event that establishes left-sided *Nodal* (Amack, 2014; Antic et al., 2010; Borovina et al., 2010; Minegishi et al., 2017; Nonaka et al., 1998, 2005; Song et al., 2010; Tabin, 2005). Although we have shown that *MZjnk1a;MZjnk1b;Zjnk2* mutants have no directional flow (Movie 6), our other mutants display reductions in nodal flow, yet directionality is maintained (Movies 1-5, 7-8). This contrasts with *MZvangl2* zebrafish and *Vangl2Vangl1* mutant mice in which normal cilia are incorrectly positioned, resulting in irregular flow and laterality disturbances (Borovina et al., 2010; Song et al., 2010). *MZmyo1d* zebrafish mutants display similar disordered nodal flow patterns, together with more gross KV defects and ultimately disruptions to left-right asymmetry (Juan et al., 2018). Loss of a single copy of *vangl2* in *MZmyo1d* mutants partially rescues nodal flow, *spaw* expression and cardiac morphogenesis in a model whereby *myo1d* and *vangl2* interact to position nodal cilia (Juan et al., 2018). This KV-specific interaction may further support the possibility that different programmes exist to generate and position nodal cilia, and that the JNK gene family is required for specification of cilia length.

Two possibilities have been suggested for the nodal flow-generated signal: morphogen flow or the two-cilia model, where nodal flow activates mechanosensory cilia (Tabin and Vogan, 2003). In support of the two-cilia model, the calcium channel *pkd2* is proposed to facilitate left-sided calcium signalling that is at least partly required for heart jogging (Yuan et al., 2015). Importantly, *pkd2* functions both autonomously and non-autonomously in KV development (Jacinto et al., 2021). This highlights candidly that, although the most overt phenotype of loss of *jnk1* and/or *jnk2* activity may be shorter nodal cilia, JNK genes may act at multiple stages of KV function, such as generation of cilia motility or sensation of nodal flow, or may also function outside KV in other tissues. Interestingly, our analysis in *MZjnk3* mutants (and also *MZjnk2* mutants), has shown that reduction of KV flow to less than 25% of wild-type levels is still sufficient to maintain left-sided *spaw* expression (Fig. 7). This indicates that either there is a threshold of KV flow required for KV function to establish the left signal and that this threshold is much lower than that generated in wild-type embryos, or that left-right axis establishment has a high degree of redundancy with other mechanisms, which may lie outside KV (Fig. S6). There is some evidence for a minimal flow threshold within the Node: as few as two motile cilia have been shown to be sufficient to generate a left-sided *Nodal* signal in mice (Shinohara et al., 2012), whereas the minimal number of functional cilia in zebrafish required for correct organ placement has been suggested to be 29 out of ∼200 (Sampaio et al., 2014). The shortening of the *lft1* expression in *jnk1* and *jnk2* mutants further supports that these genes may act not only in the KV, but also potentially downstream of *spaw* (Smith et al., 2011), possibly through a cilia-dependent mechanism at the midline (Shylo et al., 2018 preprint).

Our work supports an emerging viewpoint that certain organs possess intrinsic mechanisms that drive asymmetric morphogenesis. Loss of *spaw* does not lead to a loss of heart looping and a dextral bias is still maintained (Noël et al., 2013). Similarly, in mice, *Nodal* is dispensable for the morphogenesis of the heart tube itself (Desgrange et al., 2020; Le Garrec et al., 2017). This study, together with others, supports potential organ-specific mechanisms by demonstrating a disconnection between mesoderm and endodermal laterality (Fig. 7, Fig. S6) (Hochgreb-Hägele et al., 2013; Lopes et al., 2010; Noël et al., 2013; Sampaio et al., 2014). We have shown that *MZjnk3* embryos initially establish a left-right axis and a robust midline, and that heart jogging is normal; yet a significant proportion of embryos develop bilateral guts (Figs 7 and 8), which is a similar organ laterality phenotype to that observed in *deltaD* and *lamb1a* mutants (Hochgreb-Hägele et al., 2013; Lopes et al., 2010).

Together with left-right asymmetry, deposition of ECM components, such as laminins, and their turnover by matrix metalloproteinases, which is regulated by the transcription factor *hand2*, are necessary for asymmetric gut looping (Hochgreb-Hägele et al., 2013; Yin et al., 2010). *hand2* mutants have bilateral guts arising from failure in the necessary asymmetric cell rearrangements of the LPM (Yin et al., 2010). It is therefore tempting to speculate that *jnk3* may have a role in regulating *hand2* expression, which, coupled with our previous report of a role for *jnk1a* in regulating *hand2* expression in cardiac progenitors (Santos-Ledo et al., 2020), may suggest JNK family members have organ-specific or potentially germ layer-specific roles in regulating *hand2* activity. Additionally, the single *JNK* gene in *Drosophila* (*basket*, *bsk*) is required for anterior midgut looping (Taniguchi et al., 2007), which may suggest a partially conserved role. A further explanation for the *MZjnk3* phenotype is suggested by the interesting observation that the severity in reduction of nodal flow in the KV appears to be linked to the impact on endodermal laterality (Sampaio et al., 2014). This would suggest that a KV-dependent *spaw*-independent mechanism functions to promote gut looping morphogenesis.

Bilateral *pitx2c* expression in *MZjnk3* mutants, despite normal *spaw* and *lft1* expression, is reminiscent of exposure of embryos to retinoic acid (Tsukui et al., 1999), possibly supporting crosstalk between left-right and antero-posterior axis establishment (Kawakami et al., 2005). Our data could also suggest that there may be an alternative, late-acting midline barrier that is defective in *MZjnk3* mutants. Another possibility may be that in *MZjnk3* mutants, the right LPM is receptive to *nkx2.5* binding of the *pitx2* left side enhancer (ASE) (Shiratori et al., 2001) independently of right-sided *spaw* activity, allowing maintenance of bilateral *pitx2c*. However, the specific role that *pitx2* plays in zebrafish is unclear, as loss of *pitx2* does not impact heart or gut looping (Ji et al., 2016). Furthermore, *Pitx2* mutant mice have been reported to have initially normal organ looping but develop more-complex heart defects that may be independent of global left-right asymmetry (Ai et al., 2006; Gage et al., 1999; Lin et al., 1999; Lu et al., 1999). These observations may suggest that the endodermal phenotype in *MZjnk3* is independent of *pitx2*c. A further characterisation of brain asymmetry may also shed light on whether there is a potentially ectoderm-specific mechanism that promotes asymmetry of the habenulae (Gamse et al., 2003) or whether this is tightly coupled to asymmetric *spaw* expression in the embryo.

From these observations, it is clear that multiple pathways function during left-right axis establishment, although their hierarchy remains unresolved, exemplified in zebrafish by a *pitx2*-independent KV function-dependent cascade, for which *elovl6* is a known gene (Ji et al., 2016). Several other mechanisms, independent of nodal flow, have been proposed to establish left-right asymmetry (Levin, 2003; Tabin, 2005), possibly as early as the first cell division. Injection of dextran into one cell at the two-cell stage in zebrafish results in a lineage labelling of one side of the embryo (Noël et al., 2013), whereas, in *Xenopus*, abolishment of asymmetric localisation of 14-3-3E during the first cell division results in laterality disturbances (Bunney et al., 2003). Second, a mutation in *atp1a1a.1*, a component of the Na^+^/K^+^ transporter, presents with laterality defects, but KV structure and function appear normal (Ellertsdottir et al., 2006). Inhibition of the Na^+^/K^+^ pump with the chemical inhibitor Ouabain between 3 and 11 hpf in zebrafish also results in laterality defects (Ellertsdottir et al., 2006), and may define a critical time window because later treatment, between 10 and 13 hpf, results in KV morphogenesis defects (Juan et al., 2018), possibly suggesting that early activity of this ion pump is important for left-right asymmetry at least functioning in parallel with Nodal signalling (Fig. S6, green). Finally, JNK genes may also function outside KV to repress a right-sided factor, possibly of the *Snail* family (Isaac et al., 1997). However, the existence and role of such a factor remains controversial (Castroviejo et al., 2020; Ocaña et al., 2017; Tessadori et al., 2020). Organ asymmetry remains highly stereotypical in *MZjnk1a;MZjnk1b;Zjnk2* mutants, despite a loss of nodal flow (Movie 6) and disruption to *spaw* expression (Fig. 6A), suggesting that nodal flow is not the crucial symmetry-breaking event because other mechanisms recover initially defective cues in the early embryo (Fig. S6). Further characterisation of left-right patterning in the heart prior to jogging using markers such as *lft2* and *bmp4* (Chocron et al., 2007; Smith et al., 2008; Veerkamp et al., 2013) may uncover whether disruption to asymmetric *spaw* is recovered.

Examination of a repertoire of vertebrate model organisms highlights that nodal- and cilia-independent mechanisms may be commonplace but not yet fully elucidated (Hamada and Tam, 2020). Whereas zebrafish, *Xenopus* and mouse possess a ciliated LRO (left-right organiser), there are no luminally positioned motile cilia in the chick LRO (Hensen's node) and loss of *C2Cd3*, a gene essential for ciliogenesis, does not result in disruption to laterality (Chang et al., 2014), instead asymmetric cell movements have been proposed to break bilateral symmetry (Cui et al., 2009; Gros et al., 2009). Furthermore, the observations regarding nodal cilia have been extended to pig embryos, with the suggestion that the node is not large enough to generate fluid flow or that it may not even exist (Gros et al., 2009). Comparisons with non-vertebrate model organisms may prove to be insightful, exemplified by the role of *myo1D* in establishment of laterality in both *Drosophila* and zebrafish (Juan et al., 2018).

Both genetic and cell-based studies have suggested that JNK family members are key components of the PCP pathway (Boutros et al., 1998; Kim and Han, 2005). However, many of these have examined only a single member of the JNK family, used pharmacological methods or injected antisense-morpholino oligonucleotides, rather characterising stable mutant lines. Although unable to examine total JNK nulls due to our *jnk2* and *jnk3* alleles being generated in *trans*, this and our previous study (Santos-Ledo et al., 2020) have failed to reveal even a mild convergent-extension phenotype in JNK mutants, the hallmark of PCP signalling disturbances (Jessen et al., 2002; Park and Moon, 2002; Solnica-Krezel et al., 1996). Furthermore, we observed no PCP-related node abnormalities, such as mal-positioning or mal-distribution of cilia (Borovina et al., 2010; Wang et al., 2011). Thus, it is possible that JNK activity is not a definitive requirement for PCP signalling. Supporting this, combinatorial JNK mouse mutants, or treatment of embryos heterozygous for PCP mutations with a JNK inhibitor, do not display defects in convergent extension (Kuan et al., 1999; Ybot-Gonzalez et al., 2007). Furthermore, although suppression of JNK activity in *Drosophila* is able to rescue defective PCP signalling, loss of JNK activity alone produces only a subtle PCP phenotype in the eye (Boutros et al., 1998; Paricio et al., 1999) and in *Xenopus* explants, *JNK1* is not sufficient to regulate Wnt5a-driven convergent-extension (Yamanaka et al., 2002). However, in many of these experimental contexts, removal of kinase activity upstream of JNK signalling, such as *misshapen/TNIK* (Köppen et al., 2006; Paricio et al., 1999) or *MKK7* (Yamanaka et al., 2002), does result in classical PCP phenotypes. In summary, this suggests that, although JNK activity is a readout of active PCP signalling (Boutros et al., 1998; Li et al., 1999; Moriguchi et al., 1999), the activity of JNK itself may not be crucial for the generation of planar cell polarity and that upstream factors may be more important (Paricio et al., 1999; Yamanaka et al., 2002) or might show redundancy with JNK genes.

Establishment of laterality is proposed to be a sequential process, through an initial symmetry-breaking event commonly thought to be at the node, which is amplified to a *Nodal* homolog during early development and relayed subsequently through genes such as *Pitx2* to the organ anlagen to drive asymmetric morphogenesis. In this study, we have characterised the role of the JNK family members in the formation, function and downstream effects of nodal cilia in zebrafish. We have shown that *jnk1/jnk2* function to specify nodal cilia length, promote nodal flow and establish the *lft1* midline barrier, but that this is dispensable for the correct establishment of organ asymmetry and suggests other node-independent mechanisms are able to recover this early phenotype (Fig. S6). We have also identified a novel later requirement for *jnk3* to maintain lateralised *pitx2c* expression that may either directly or indirectly promote asymmetric morphogenesis of the endoderm. Together, this demonstrates that multiple mechanisms, both JNK dependent and independent act redundantly at different stages during vertebrate axis establishment, ensuring robust asymmetric organ morphogenesis.

## MATERIALS AND METHODS

### Zebrafish handling and maintenance

The following previously described zebrafish lines used in this study were wild type (AB), *jnk1a^n2^* (*mapk8a*) and *jnk1b^n3^* (*mapk8b*) (Santos-Ledo et al., 2020). All procedures and experimental protocols were carried out in accordance with UK Home Office and Newcastle University (Project Licence P25F4F0F4). Embryos were obtained from natural pairwise mating and reared in standard conditions. Embryos were raised in Embryo Medium (E3) at 28.5°C and staged according to Kimmel et al. (1995).

### Generation of *jnk2* and *jnk3* mutants

*jnk2* (*mapk9*, ENSDARG00000077364, ZDB-GENE-091117-28) and *jnk3* (*mapk10*, ENSDARG00000102730*,* ZDB-GENE-051120-117) zebrafish mutants were generated using CRISPR-Cas9-mediated mutagenesis (Hwang et al., 2013). gRNAs were identified using CrisprScan (Moreno-Mateos et al., 2015). gRNAs and Cas9 RNA were synthesized according to previously published protocols (Gagnon et al., 2014) and injected at the one-cell stage into embryos obtained from an in-cross of wild-type (AB) adults (F0 generation). The 20 bp deletion in Exon 3 of *jnk2* (allele designation *mapk9^n4^*) was generated using the single gRNA 5′-ATTTAGGTGACACTATAGCAATCTTCACATCCAGGACGTTTTAGAGCTAGAAATAGCAAG-3′ and genotyped using forward (5′-TTAAAGGGGATTGAGGAACAAA-3′) and reverse (5′-GTTAAGGGGACGTACGTTCTTG-3′) primers in a standard GoTaq G2 (Promega M784B) PCR with an annealing temperature of 58°C and 34 cycles. The mutation destroys a DdeI restriction enzyme site (New England Biolabs R1075). The 4 bp deletion in exon 5 of *jnk3* (allele designation *mapk10^n5^*) was generated using the single gRNA 5′-ATTTAGGTGACACTATAGGCCACATTTCTGTCCAGGAGTTTTAGAGCTAGAAATAGCAAG-3′ and genotyped using forward (5′-AATTCCCATCTTGTGTTTCAGG-3′) and reverse (5′-TTTTGGGGAAAACCTGACTCTAA-3′) primers in a standard GoTaq G2 PCR with an annealing temperature of 58°C and 34 cycles. The mutation destroys a BstNI restriction enzyme site (New England Biolabs R1068). F0 adults identified as carrying desired mutations were outcrossed to wild-type animals prior to generation of homozygous mutant lines. Where lines were derived from multiple rounds of in-crossing, a minimum of six different breeding pairs were used to establish the next generation. Reduction in mRNA levels in *MZjnk2* or *MZjnk3* mutants was confirmed by RT-PCR using primers and protocols previously described (Santos-Ledo et al., 2020).

### Immunohistochemistry

Fixed embryos were serially rehydrated from 100% methanol into PBST [0.2% Tween-20 (Sigma P2287) in 1× PBS (Oxoid BR0014G)], washed three times in 0.2% Triton-X (Sigma T8787) in 1× PBS (PBSTx) and then incubated in blocking buffer for 1 h at room temperature [5% sheep serum (Gibco, 16070-096), 10 mg/ml bovine serum albumin (A2153, Sigma) and 1% DMSO (D4540, Sigma) in PBSTx] for 1 h at room temperature before an overnight incubation at 4°C with gentle agitation with the following primary antibodies: mouse anti-acetylated tubulin (T6793, Sigma, 1:500) and rabbit anti-PKC ζ (aPKC) (sc-216, Santa Cruz, 1:400) in blocking buffer (Amack et al., 2007). The next day, embryos were rinsed extensively in PBSTx, before a further overnight incubation at 4°C with gentle agitation with the following secondaries: Alexa488-conjugated donkey anti-mouse (Invitrogen A21202, 1:200) and Alexa647-conjugated donkey anti-rabbit (Invitrogen A31573, 1:200) in blocking buffer. Embryos were washed extensively on day three before dissection or embedding. Representative images of Kupffer's vesicle were taken using a Nikon A1 inverted confocal using a 40× objective.

### Quantification of cilial length

Embryos were embedded in 1.5% low melting agarose (ThermoFisher Scientific R0801) in PBS and imaged in an Axiotome microscope using a 40× objective. An image stack was recorded and used for quantification. Stacks were opened in Fiji where the number of cilia and their position relative to the anterior and posterior parts of Kupffer's vesicle were quantified (Wang et al., 2011). The *xy* coordinates of all cilia were recorded in every slice. The total length of the cilia was obtained as previously described (Dummer et al., 2016). The *xy* coordinates were translated to µm assuming the following equivalencies: 1 pixel=0.1623 µm, 1 slice=0.4 µm.

### Quantification of fluid flow in Kupffer's vesicle by microbead injection

Analysis of nodal flow was carried out as previously described (Wang et al., 2013), using TransFluoSpheres beads of 1 µm diameter (Invitrogen T8880). High-speed movies were acquired using an Axiotome microscope with either a 40× or 60× objective attached to an IMPERX high speed camera with Video Savant 4.0 software (Multipix, UK). The frame rate of acquired movies was 207 frames per second. Stacks were imported into Fiji (Schindelin et al., 2012), the notochord oriented to the top of the image and the quadrants indicated. Every fifth frame representing ∼0.0125 s were used to track bead speed by the Manual Tracking plug-in. Beads were tracked that remained in the focal plane of the movie for ≥50 frames. Average speeds of beads were then calculated for the duration of tracking (Wang et al., 2011, 2013).

### mRNA *in situ* hybridisation

Embryos older than 24 hpf for use in *in situ* hybridisation were transferred into E3 medium containing 0.003% 1-phenyl 2-thiourea (PTU, Sigma P7629) to inhibit pigment formation and aid imaging. Embryos were fixed overnight in 4% paraformaldehyde (PFA, P6148, Sigma) in 1× phosphate-buffered saline, washed three times in PBST for 5 min at room temperature and then serially washed into 100% methanol for long-term storage at −20°C. Whole-mount *in situ* hybridisation was carried out according to standard protocols (Thisse and Thisse, 2008). The following, previously published probes were used: *dand5* (Hashimoto et al., 2004), *spaw* (Long et al., 2003), *myl7* (Yelon et al., 1999), *foxa3* (Odenthal and Nüsslein-Volhard, 1998), *lft1* (Bisgrove et al., 1999) and *pitx2c* (Yan et al., 1999).

### Quantification of *lft1* domain

*In situ* hybridisation images were imaged laterally using a Zeiss AxioPlan. Images were pooled and made unidentifiable using Image J Blind_Analysis plug-in as previously described (Derrick et al., 2021) and imported into Fiji (Schindelin et al., 2012). Using the Freehand Line tool, the length of the domain with continuous expression was measured from the caudal tip to the anterior extreme. Embryos without any visible *lft1* expression were discounted from analysis.

### Statistical analysis

No statistical tests were used to formally predetermine sample size, but the number of biological replicates were based on published studies and defined *a priori*. For all experiments where n was based on the individual embryo (e.g. KV flow), they were derived from at least three different clutches from distinct breeding pairs. For population analysis of laterality markers or organ asymmetry, *n* was represented by the clutch, and each clutch was derived from three distinct breeding pairs. Statistical tests were carried out in Prism (Graphpad).

## Supplementary Material

Supplementary information
