# Supplementary material for: Sequential action of JNK genes establishes the embryonic left-right axis
Source: Development. 2022 May 3;149(9):dev200136. doi: 10.1242/dev.200136 (PMC9148569; doi:10.1242/dev.200136)
Supplement: Supplementary information [file develop-149-200136-s1.pdf]

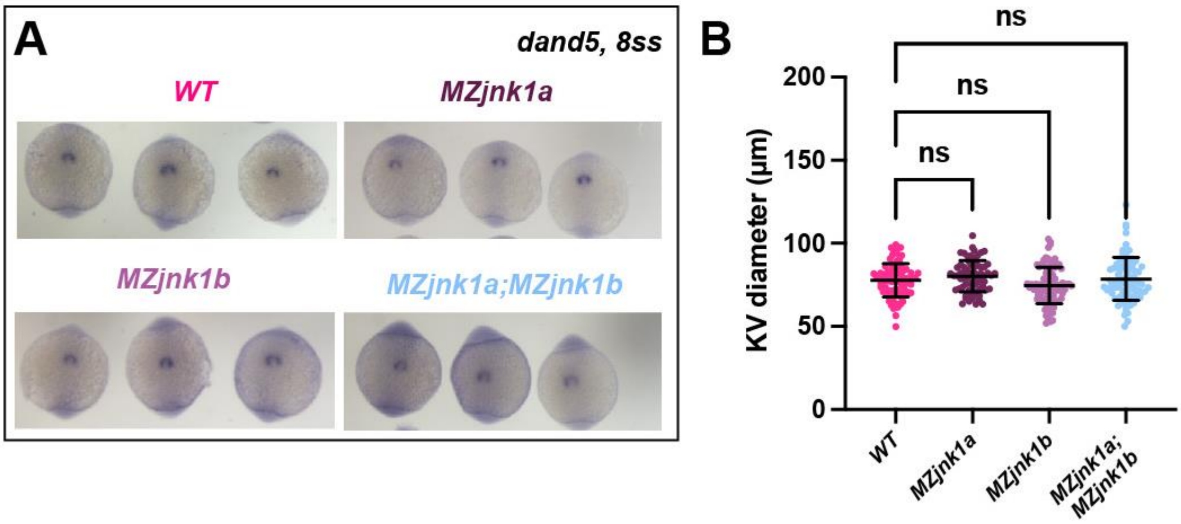

Fig. S1. Loss of *jnk1* does not impact Kupffer's Vesicle Size (A) Representative images of mRNA *in situ* hybridisation of *DAN domain family member 5* (*dand5)* at 8ss marking KV for quantification of diameter in WT, *MZjnk1a, MZjnk1b* and *MZjnk1a;MZjnk1b* embryos. **(B)** KV diameter quantification in WT, *MZjnk1a, MZjnk1b* and *MZjnk1a;MZjnk1b* embryos. KV size is not impacted by loss of *jnk1* function B: Mean ± S.D, One-way ANOVA, multiple comparisons, WT n=94, *MZjnk1a* n=68, *MZjnk1b* n=91, *MZjnk1a;MZjnk1b* n=84. A: Posterior up, left: right.

Development • Supplementary information

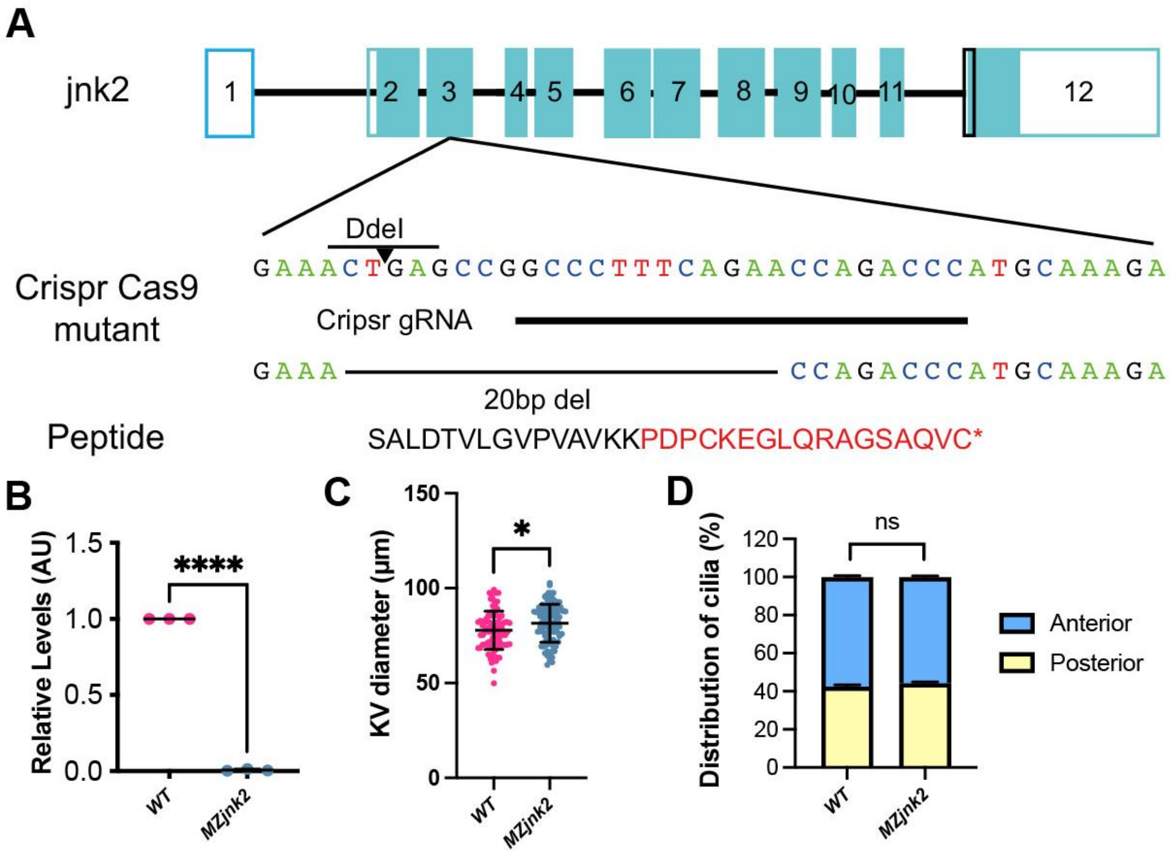

Fig. S2. Generation of *jnk2* mutants by CRISPR-Cas9 genome editing

(A) Schematic of *jnk2* (*mapk9*) gene structure based on ZDB-GENE-091117-28, white denotes untranslated region, green denotes coding sequence. *jnk2* mutants were generated using a single gRNA targeting Exon 3 (ENSDART00000112550.3, isoform 1), downstream of a DdeI restriction enzyme site. The recovered 20bp mutation destroys the DdeI restriction site, generating a frame shift, leading to 17 amino acids of nonsense and a stop codon (red). **(B)** Semi-quantitative PCR demonstrates that *MZjnk2* mutants are a complete null for *jnk2*. **(C)** KV diameter quantification in WT and *MZjnk2* embryos at 8ss. *MZjnk2* mutant embryos display a mild, yet significant increase in KV diameter. **(D)** Quantification of cilial distribution in WT control and *MZjnk2* embryos at 10ss. Antero-posterior distribution of nodal cilia is unaffected by loss of *jnk2*. B: Mean ± Standard Error Mean, Unpaired t test, n = 3. C: Mean ± S.D, Welch's t-test, *MZjnk2* n=94, WT from Figure S1B. D: Mean ± Standard Error Mean, Two-way ANOVA, *MZjnk2* n=16, WT data reproduced from Figure 1G. ns: not significant, *:p<0.05, ****: p<0.0001.

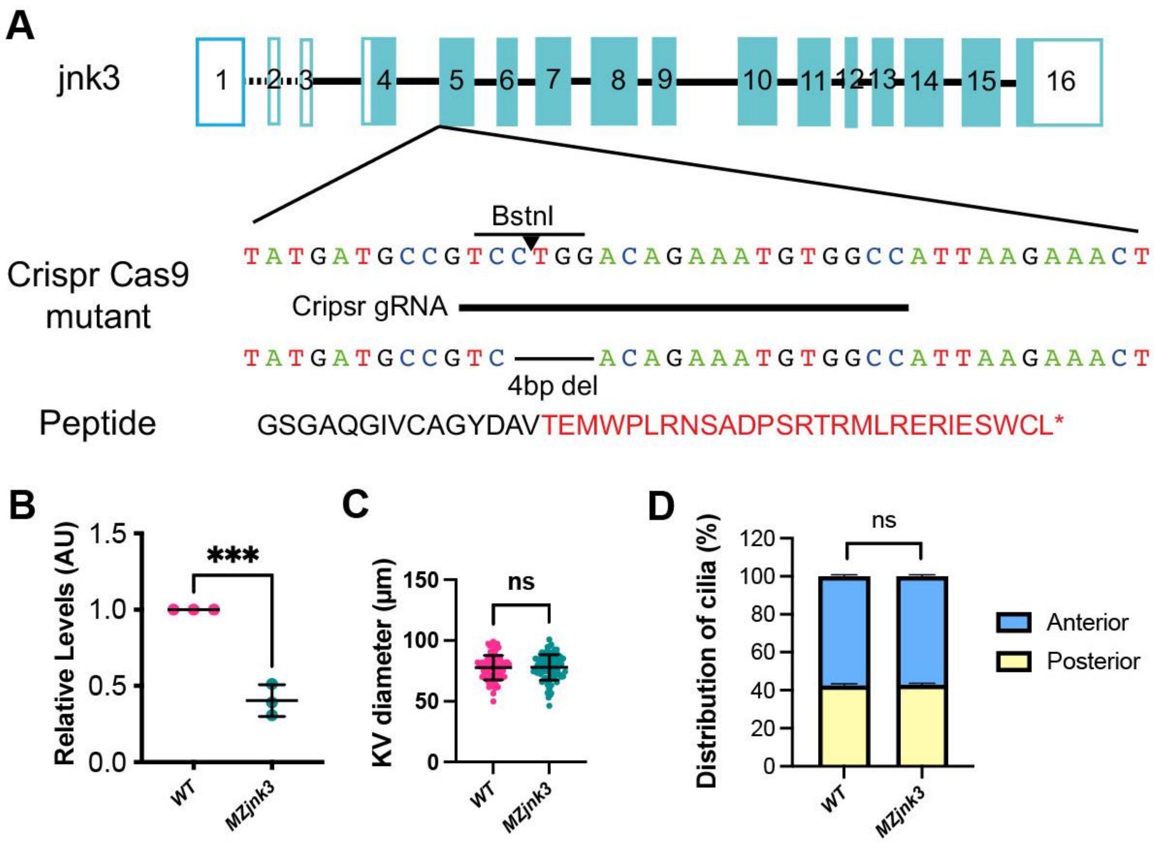

Fig. S3. Generation of *jnk3* mutants by CRISPR-Cas9 genome editing

(A) Schematic of *jnk3* (*mapk10*) gene structure based on ZDB-GENE-051120-117, white denotes UTR, green denotes coding sequence. *jnk3* mutants were generated using a single gRNA targeting Exon 5 (NM_001318318, isoform 1), downstream of a BstnI restriction enzyme site. The recovered 4bp mutation destroys the BstnI restriction site generating a frame shift, leading to 27 amino acids of nonsense and a stop codon (red). **(B)** Semi-quantitative PCR demonstrates that *MZjnk3* mutants have approximately 50% reduction in *jnk3* transcript. **(C)** KV diameter quantification in WT and *MZjnk3* embryos at 8ss. Loss of *jnk3* does not impact KV size. **(D)** Quantification of cilial distribution in WT control and *MZjnk3* embryos at 10ss. Antero-posterior distribution of nodal cilia is unaffected by loss of *jnk3*. B: Mean ± Standard Error Mean, Unpaired t test, n = 3. C: Mean ± S.D, Welch's t test, *MZjnk3* n=89, WT from Figure S1B. D: Mean ± Standard Error Mean, Two-way ANOVA, *MZjnk3* n=16, WT data reproduced from Figure 1G. ns: not significant, ***: p<0.001.

Development • Supplementary information

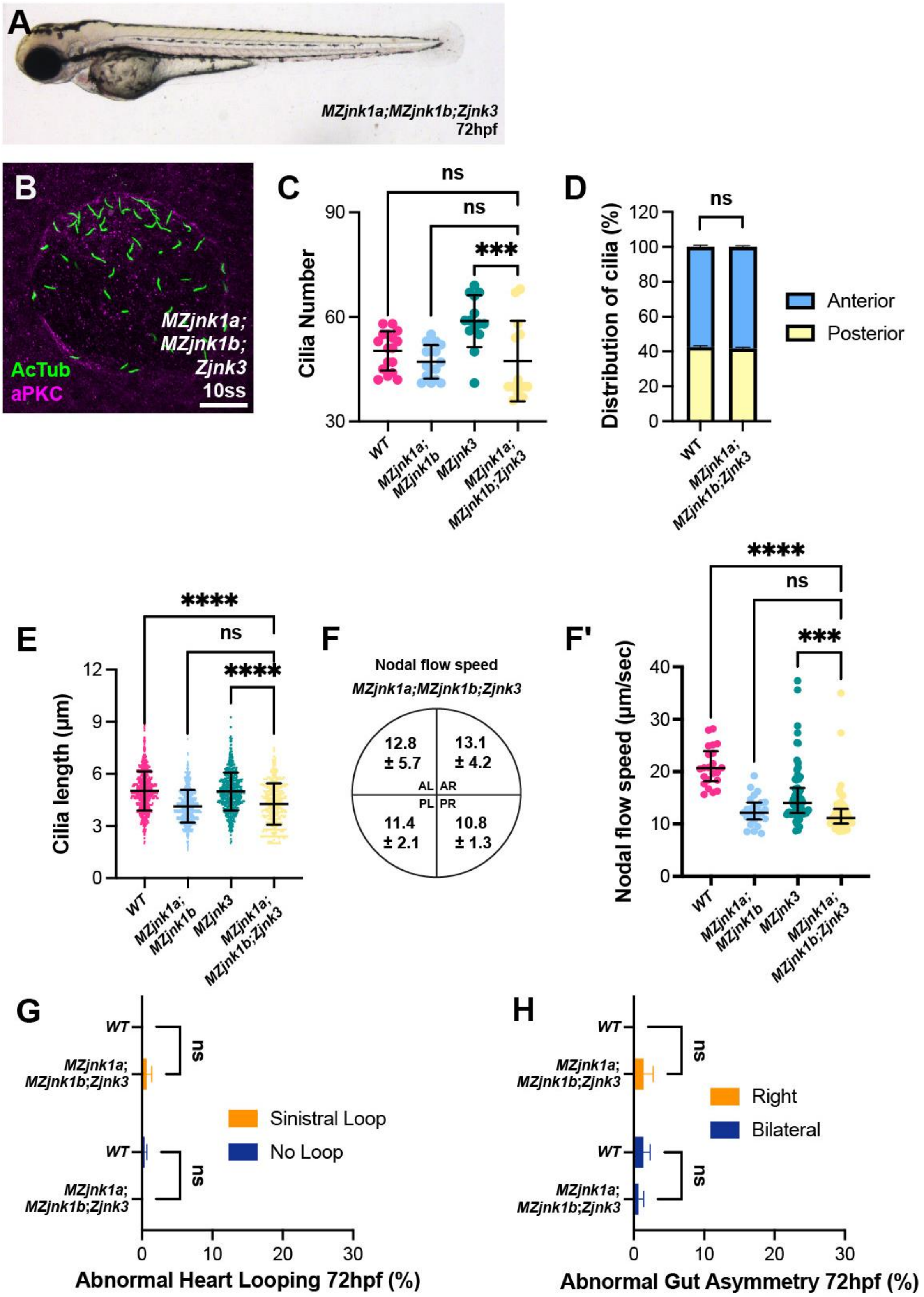

Fig. S4. *jnk3* is epistatic to *jnk1* in generating nodal flow

(A) Representative bright field image of *MZjnk1a;MZjnk1b;Zjnk3* embryo at 3dpf. **(B)** Representative image of *MZjnk1a;MZjnk1b;Zjnk3* KV at 10ss labelling Acetylated Tubulin (Green) and aPKC (Magenta). **(C)** Quantification of number of cilia in WT

Development • Supplementary information

control, *MZjnk1a;MZjnk1b*, *MZjnk3* and *MZjnk1a;MZjnk1b;Zjnk3* mutant embryos at 10ss. Loss of *jnk1* activity rescues increased cilia number in *jnk3* mutants. **(D)** Quantification of cilial distribution of in WT control and *MZjnk1a;MZjnk1b;Zjnk3* embryos at 10ss. Antero-posterior distribution of nodal cilia is unaffected by loss of *jnk3* in a *jnk1* null background. **(E)** Quantification of length of nodal cilial in WT control, *MZjnk1a;MZjnk1b*, *MZjnk3* and *MZjnk1a;MZjnk1b;Zjnk3* mutant embryos at 10ss. Loss of *Zjnk3* activity in *MZjnk1a;MZjnk1b* mutant background does not further impact length of nodal cilia. **(F-F')** Quantification of nodal flow speed in *MZjnk1a;MZjnk1b;Zjnk3* by quadrant (F) and average speed (F') between 10-14ss. Loss of *Zjnk3* activity in *MZjnk1a;MZjnk1b* mutant background does not further impact average speed of nodal flow in KV. Quantification of **(G)** heart looping and **(H)** gut looping in WT control and *MZjnk1a;MZjnk1b;Zjnk3* mutants at 72hpf. Loss of *jnk1* and *jnk3* activity does not impact organ laterality. C: Mean ± Standard Deviation, One-way ANOVA with multiple comparisons, *MZjnka1a;MZjnk1b;Zjnk3* n=13, WT and *MZjnk1a;MZjnk1b* data reproduced from Figure 1F, *MZjnk3* data reproduced from Figure 7C. D: Mean ± Standard Error Mean, Two-way ANOVA, *MZjnk1a;MZjnk1b;Zjnk3* n=15, WT data reproduced from Figure 1G. E: Mean ± Standard Deviation, One-way ANOVA with multiple comparisons, *MZjnk1a:MZjnk1b;Zjnk3* n=577, WT and *MZjnk1a:MZjnk1b* data reproduced from Figure 1H, *MZjnk3* data reproduced from Figure 7D. F': Median ± Interquartile Range, Kruskal-Wallis test with multiple comparisons, *MZjnk1a;MZjnk1b;Zjnk3* n = 63 beads across 8 embryos, WT and *MZjnk1a;MZjnk1b* data reproduced from Figure 2F, *MZjnk3* data reproduced from Figure 7E'. G: Mean ± Standard Error Mean, Two-way ANOVA comparison of left and No loop. N = 3 clutches. Minimum clutch size *MZjnk1a;MZjnk1b;Zjnk3*, n = 44, WT data reproduced from 6D. H: Mean ± Standard Error Mean, Two-way ANOVA comparison of Right and Bilateral. N = 3 clutches, same clutches as S4G. WT data reproduced from 6E. A: lateral view, anterior left, B: anterior up. Scale bar: 20µm. ns: not significant, ***: p<0.001, ****: p<0.0001. AL: anterior left, AR: anterior right, PL: posterior left, PR: posterior right.

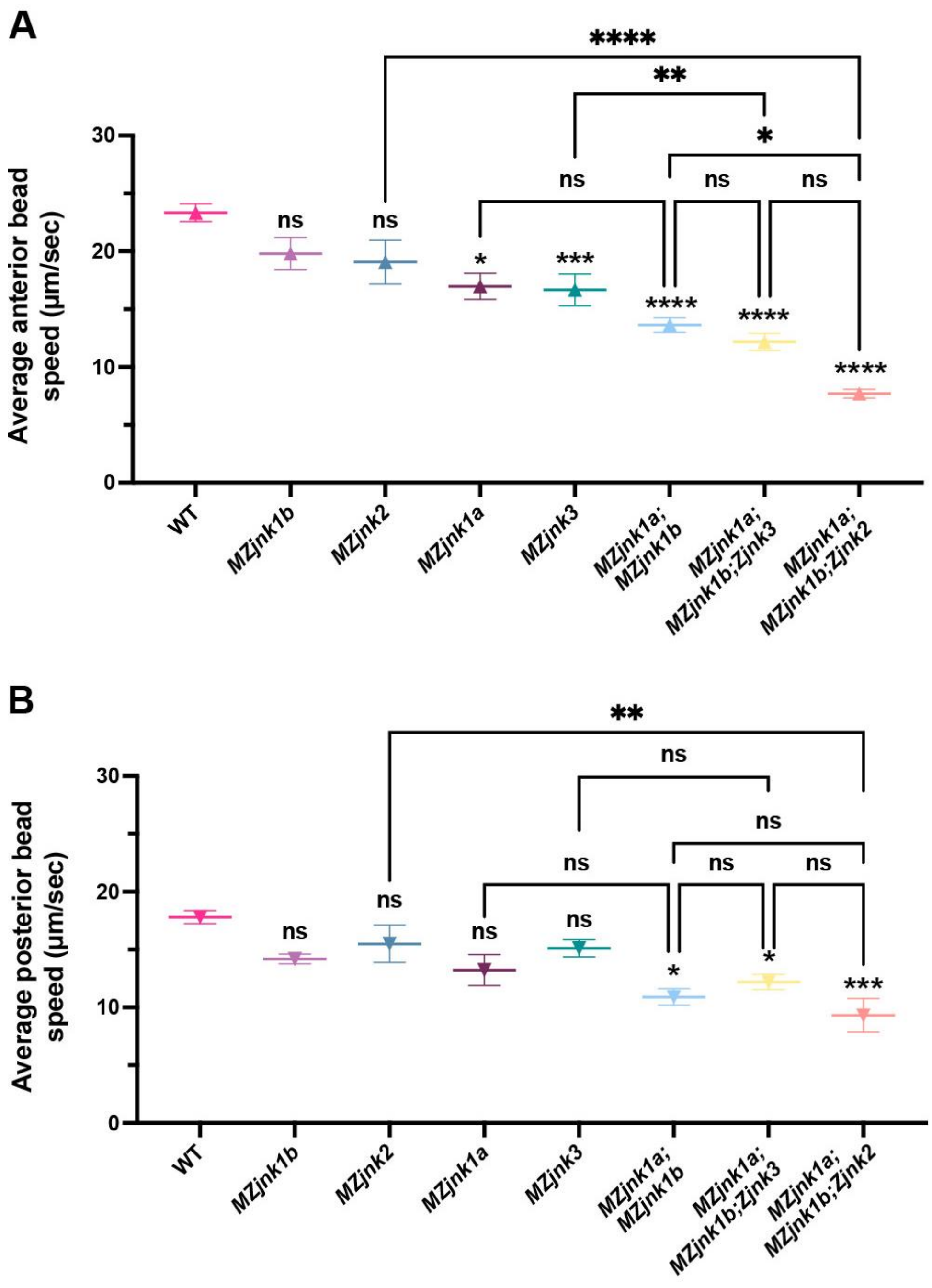

Fig. S5. Loss of *jnk* impacts nodal flow speeds in diverse ways

(A) Average speed of beads imaged in anterior compartment of KV in respective genotype, reproduced from Figures 2, 4, 5, 7 and S4. Notation directly above average values denotes statistical significance when compared to WT. Anterior nodal flow is impacted except in *MZjnk1b* or *MZjnk2* mutants. The most dramatic reduction in anterior nodal flow speed is in *MZjnk1a;MZjnk1b;Zjnk2* mutants. **(B)** Average speed of beads imaged in posterior compartment of KV in respective

Development • Supplementary information

genotype, reproduced from Figures 2, 4, 5, 7 and S4. Notation directly above average values denotes statistical significance when compared to WT. Posterior nodal flow is only significantly altered following loss of both *jnk1a* and *jnk1b*, but is not further modified by loss of *jnk2* or *jnk3*. A-B: Mean ± Standard Error Mean, One-way ANOVA with multiple comparisons ns: not significant, *: $p<0.05$, **: $p<0.01$, ***: $p<0.001$, ****: $p<0.0001$.

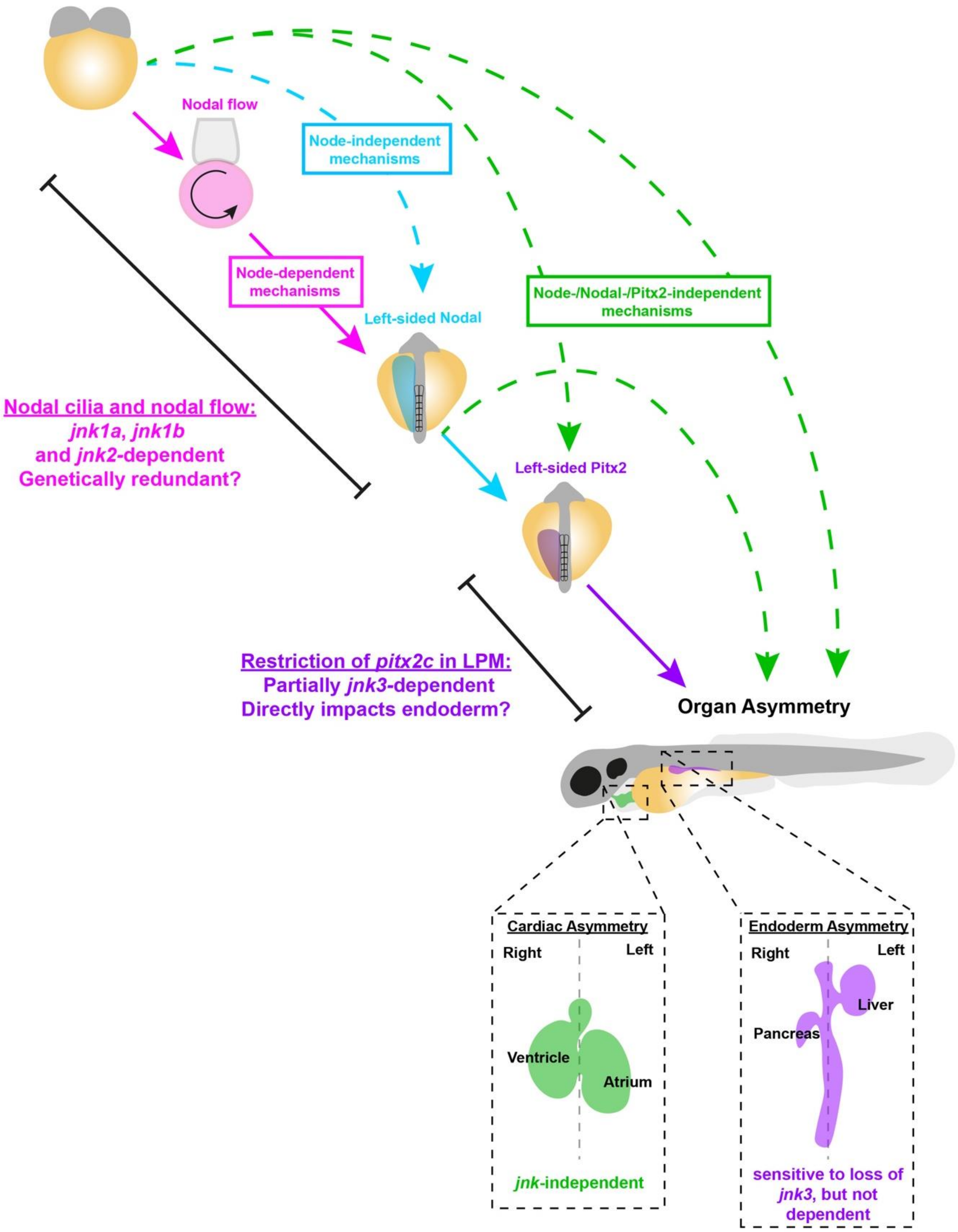

Fig. S6. The role of *jnk* genes in establishment of the left-right axis

Multiple mechanisms ensure robust asymmetric organ placement during development. An early node-dependent mechanism (magenta), requiring both *jnk1* and *jnk2* functions to specify cilia length and drive directional nodal flow. Nodal flow

Development • Supplementary information

(magenta) and Node-independent mechanisms (blue) result in asymmetric, left-side restricted *Nodal* (blue) in the left LPM. Downstream of Nodal and other early acting mechanisms, *Pitx2* expression is restricted to the left LPM, which is partially dependent on *jnk3* (purple). Pathways independent of the node, *Nodal* and *Pitx2* function also define the left-right axis in parallel and may act downstream of *Nodal* expression (green). A combination of *Nodal* and *Pitx2*-dependent and -independent pathways result in organ asymmetry. Some organs such as the heart, undergo asymmetric morphogenesis independent of node, *Nodal*, *Pitx2* or *jnk* activity (green) whilst endoderm asymmetry may be dependent on restricted *pitx2c* expression, which partially requires *jnk3* (purple) or function through *Pitx2*-independent, mechanisms which are sensitised to disruption in *MZjnk3* mutants.

Development • Supplementary information

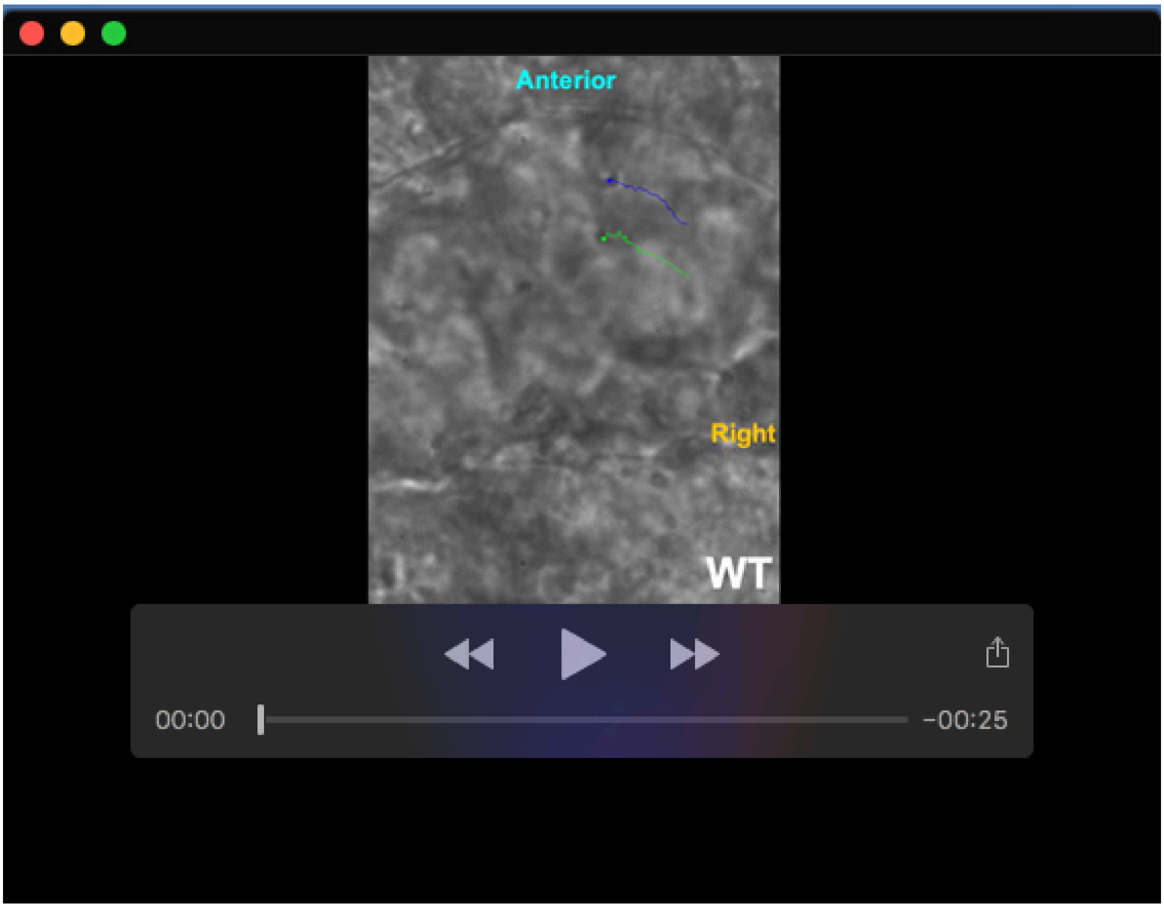

Movie 1. WT Kupffer's Vesicle

Brightfield movie of example WT control Kupffer's vesicle injected with beads for quantification of nodal flow, imaged using a 60X objective. Dark blue and green traces: Representative Anterior, red trace: Representative Posterior.

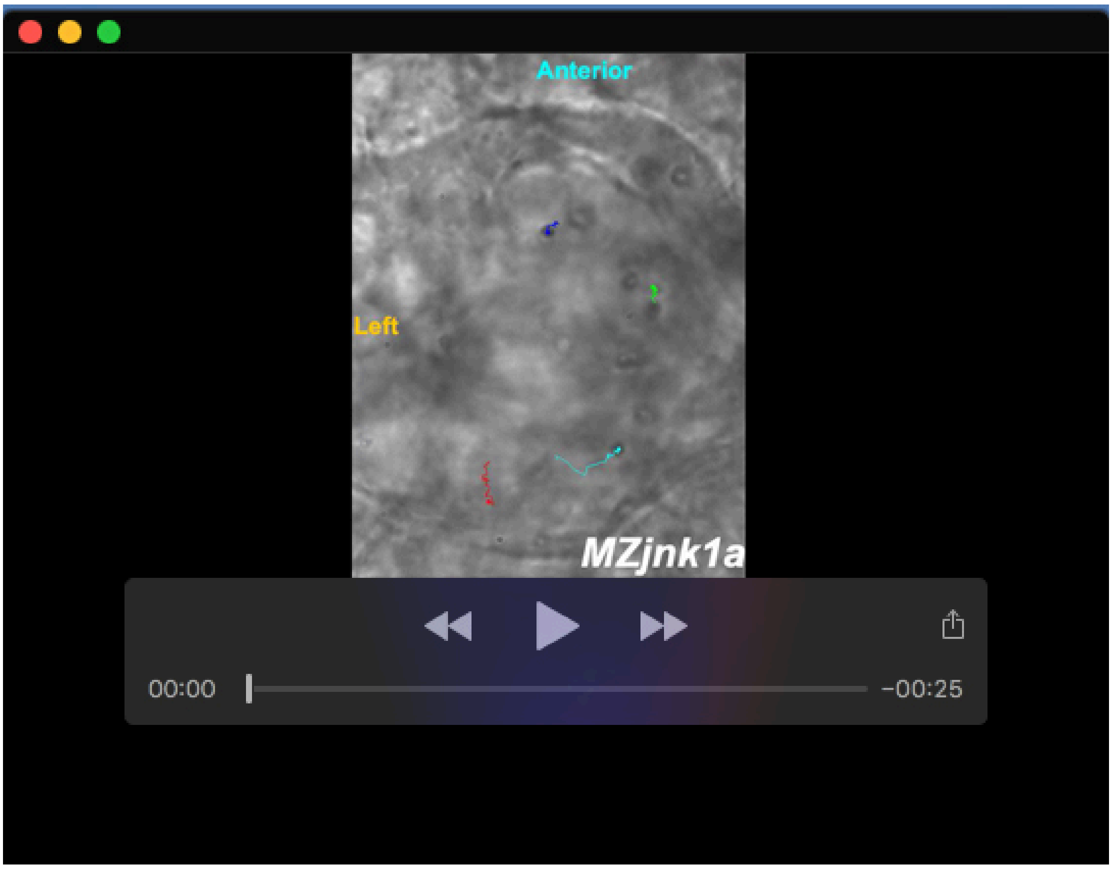

Movie 2. ***MZjnk1a*** **Kupffer's Vesicle**

Brightfield movie of example *MZjnk1a* Kupffer's vesicle injected with beads for quantification of nodal flow, imaged using a 60X objective. Dark blue and green traces: Representative Anterior, red and light blue traces: Representative Posterior.

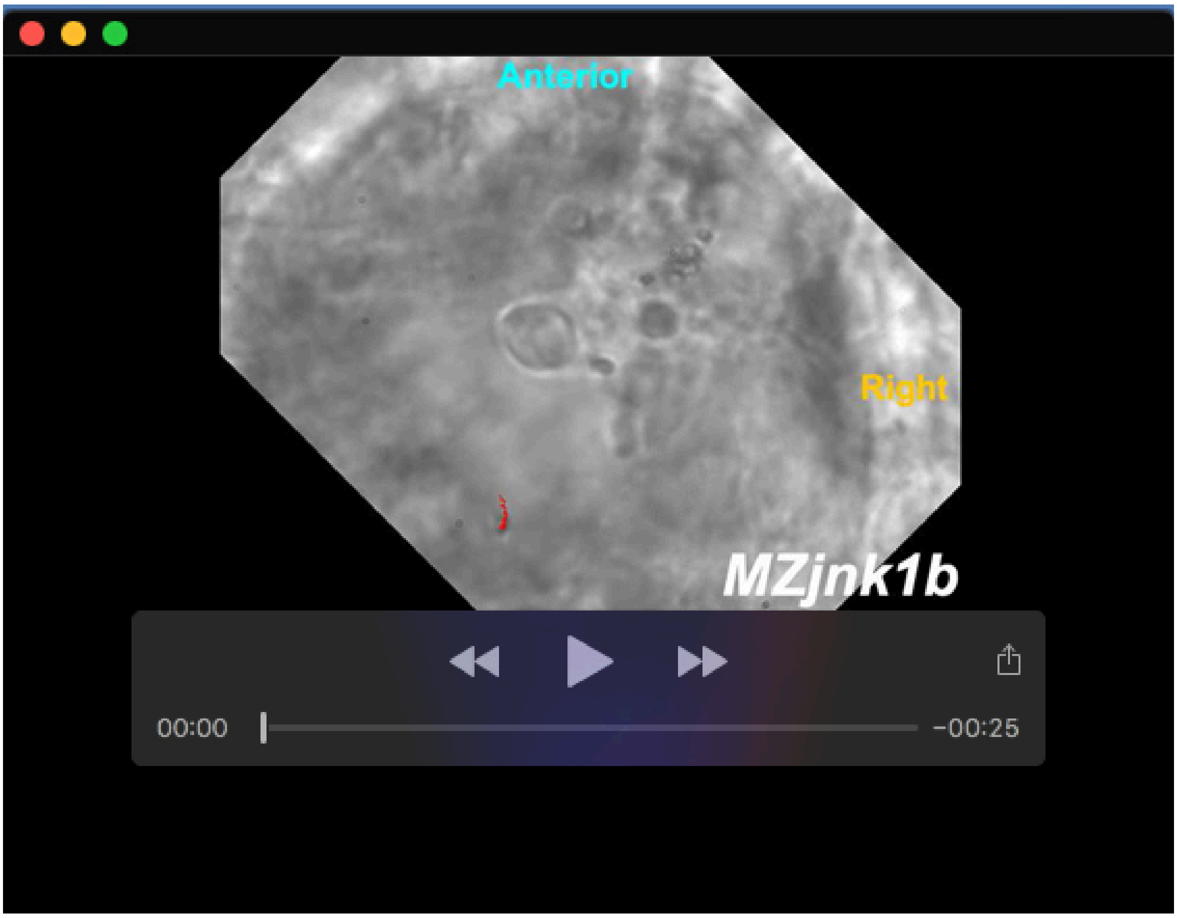

Movie 3. *MZjnk1b* Kupffer's Vesicle

Brightfield movie of example *MZjnk1b* Kupffer's vesicle injected with beads for quantification of nodal flow, imaged using a 60X objective. Dark blue and green traces: Representative Anterior, red and light blue traces: Representative Posterior.

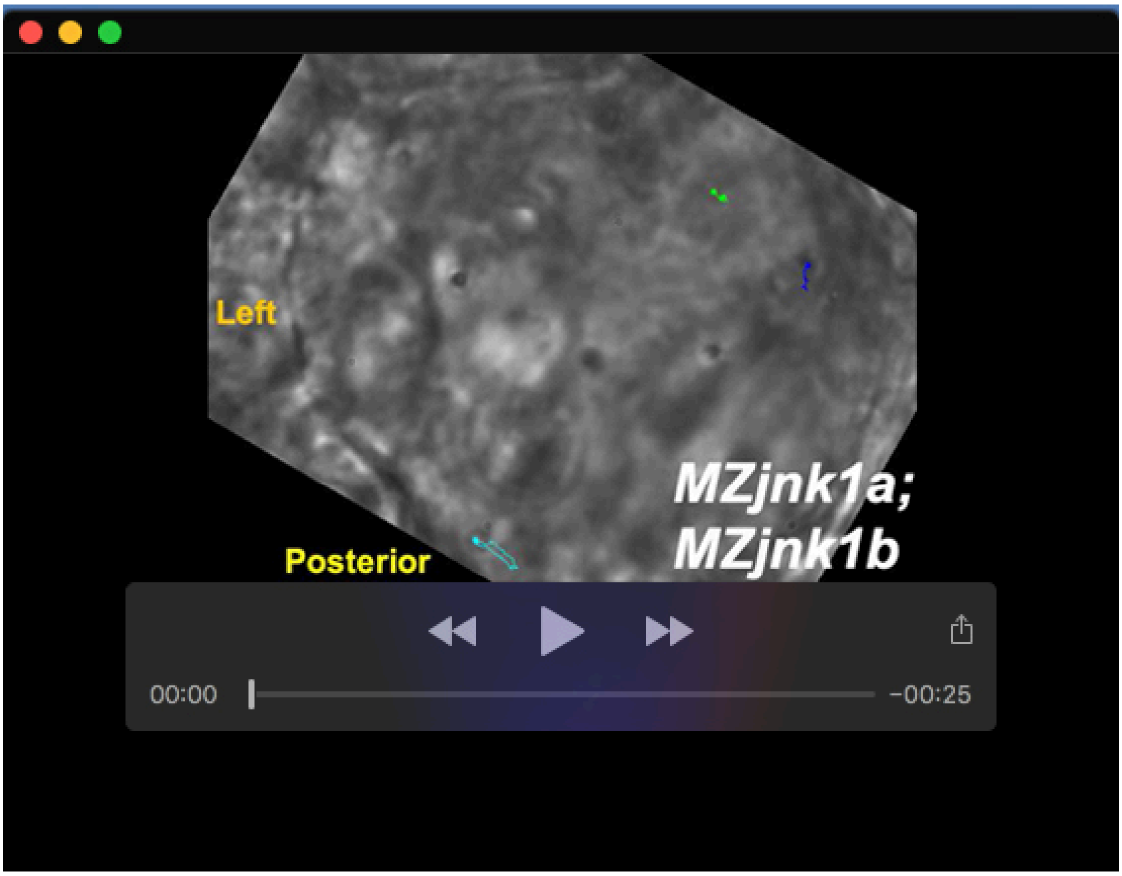

Movie 4. ***MZjnk1a;MZjnk1b*** **Kupffer's Vesicle**

Brightfield movie of example *MZjnk1a;MZjnk1b* Kupffer's vesicle injected with beads for quantification of nodal flow, imaged using a 60X objective. Dark blue and green traces: Representative Anterior, red and light blue traces: Representative Posterior.

Development • Supplementary information

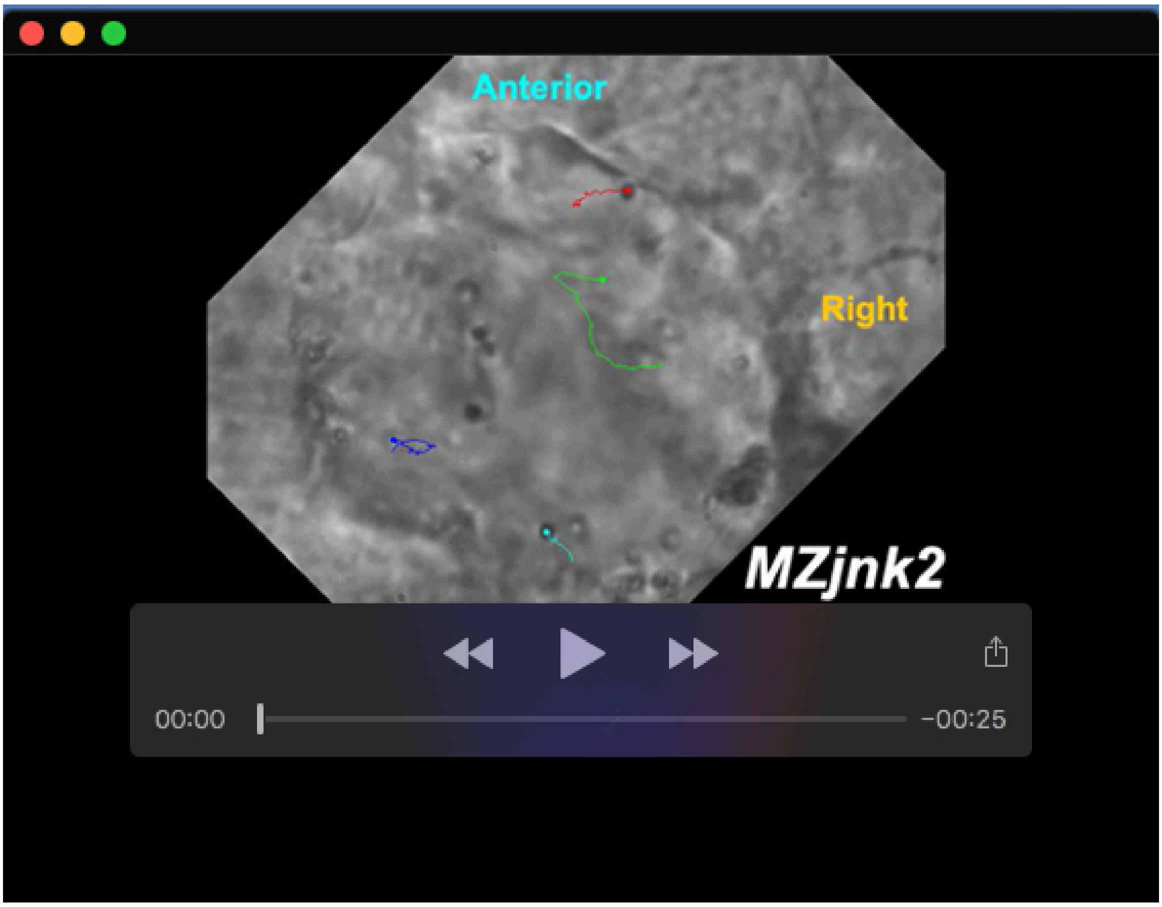

Movie 5. *MZjnk2* Kupffer's Vesicle

Brightfield movie of example *MZjnk2* Kupffer's vesicle injected with beads for quantification of nodal flow, imaged using a 60X objective. Dark blue and green traces: Representative Anterior, red and light blue traces: Representative Posterior.

Development • Supplementary information

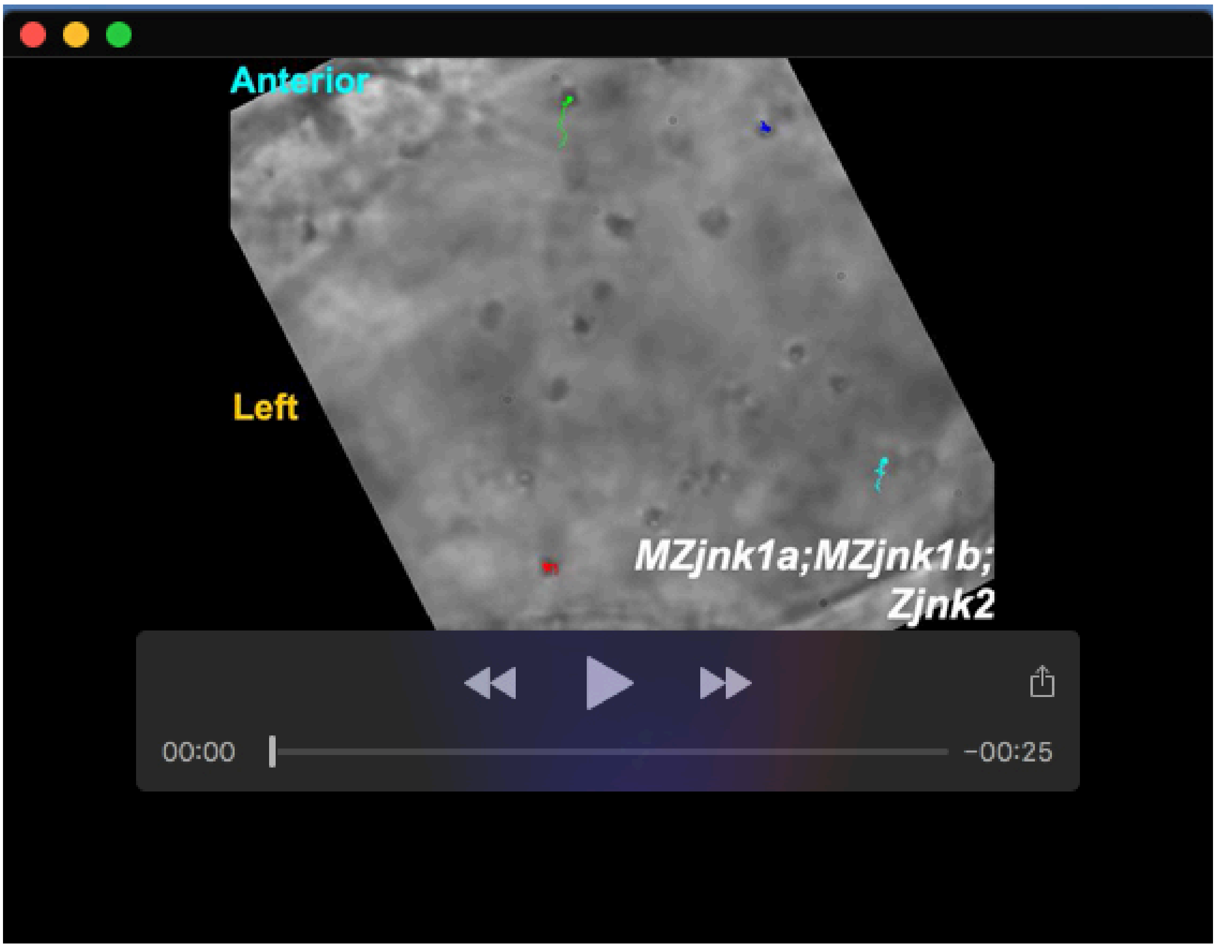

Movie 6. ***MZjnk1a;MZjnk1b;Zjnk2*** **Kupffer's Vesicle**

Brightfield movie of example *MZjnk1a;MZjnk1b;Zjnk2* Kupffer's vesicle injected with beads for quantification of nodal flow, imaged using a 60X objective. Nodal flow is dramatically reduced, with beads showing very little directional movement. Dark blue and green traces: Representative Anterior, red and light blue traces: Representative Posterior.

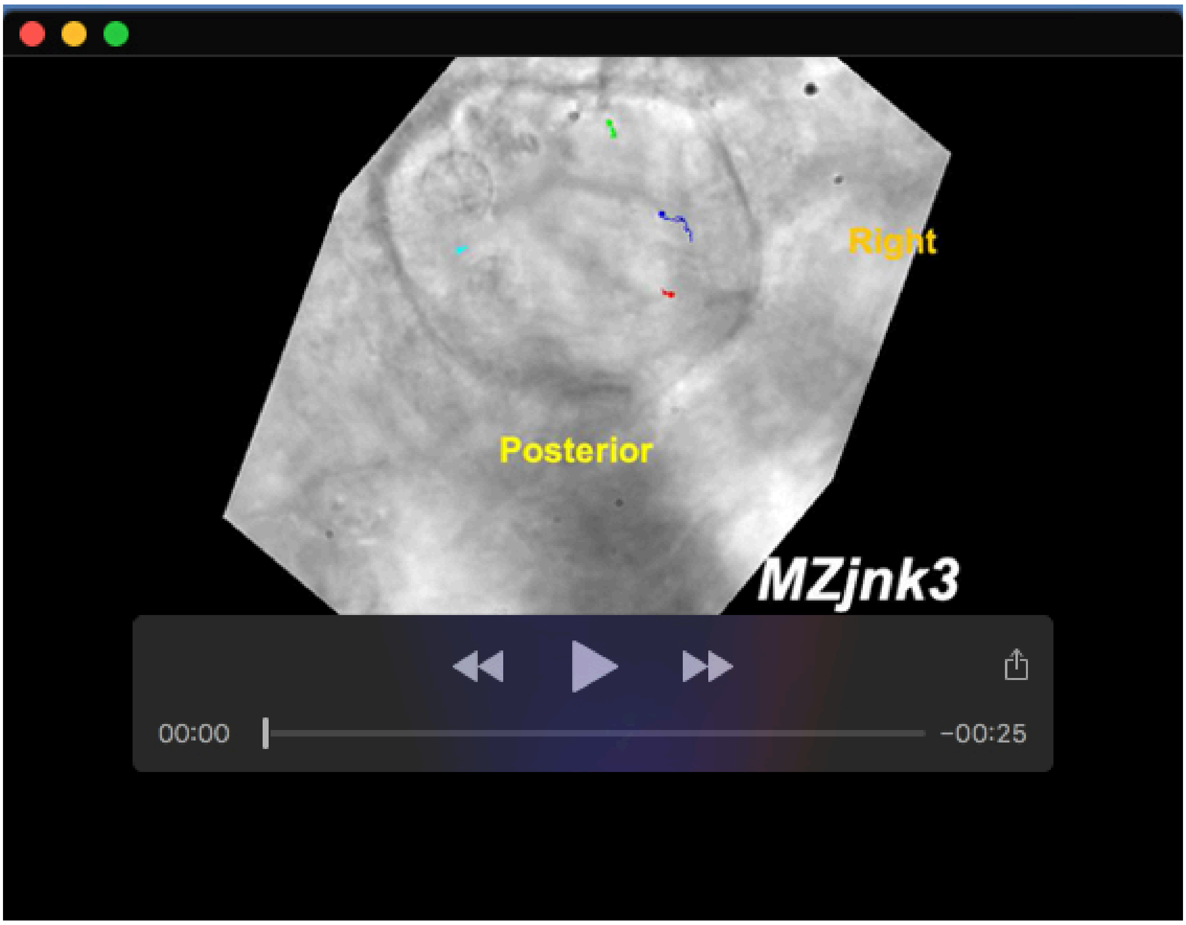

Movie 7. *MZjnk3* Kupffer's Vesicle

Brightfield movie of example *MZjnk3* Kupffer's vesicle injected with beads for quantification of nodal flow, imaged using a 40X objective. Dark blue and green traces: Representative Anterior, red and light blue traces: Representative Posterior.

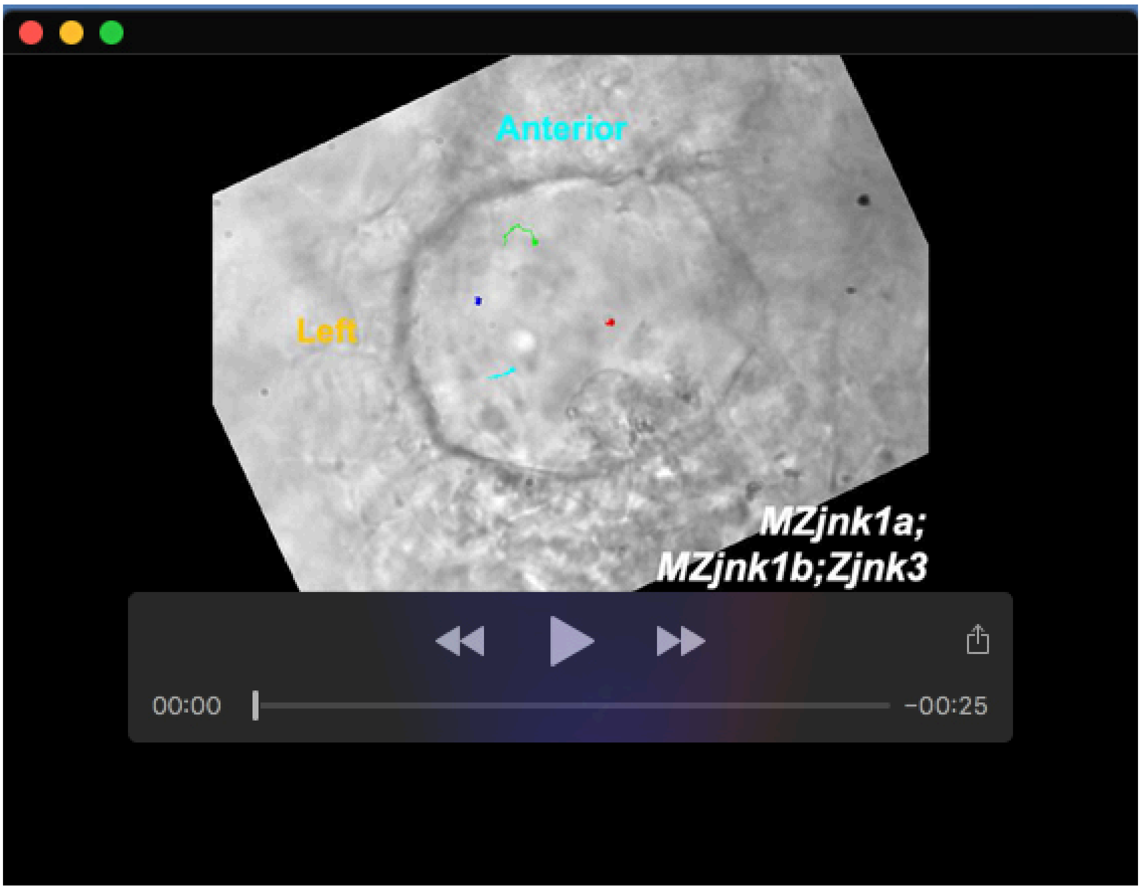

Movie 8. ***MZjnk1a;MZjnk1b;Zjnk3*** **Kupffer's Vesicle**

Brightfield movie of example *MZjnk1a;MZjnk1b;Zjnk3* Kupffer's vesicle injected with beads for quantification of nodal flow, imaged using a 40X objective. Dark blue and green traces: Representative Anterior, red and light blue traces: Representative Posterior.
